# A widespread peroxiredoxin-like domain present in tumor suppression- and progression-implicated proteins

**DOI:** 10.1186/1471-2164-11-590

**Published:** 2010-10-21

**Authors:** Krzysztof Pawłowski, Anna Muszewska, Anna Lenart, Teresa Szczepińska, Adam Godzik, Marcin Grynberg

**Affiliations:** 1 Institute of Biochemistry and Biophysics, PAS, Pawińskiego 5A, 02-106 Warsaw, Poland; 2 Nencki Institute of Experimental Biology, PAS, Pasteura 3, 02-093 Warsaw, Poland; 3 Sanford|Burnham Medical Research Institute, 10901 N. Torrey Pines Rd., La Jolla, CA 92037, USA; 4 Warsaw University of Life Sciences, Nowoursynowska 166, 02-787 Warsaw, Poland

## Abstract

**Background:**

Peroxide turnover and signalling are involved in many biological phenomena relevant to human diseases. Yet, all the players and mechanisms involved in peroxide perception are not known. Elucidating very remote evolutionary relationships between proteins is an approach that allows the discovery of novel protein functions. Here, we start with three human proteins, SRPX, SRPX2 and CCDC80, involved in tumor suppression and progression, which possess a conserved region of similarity. Structure and function prediction allowed the definition of P-DUDES, a phylogenetically widespread, possibly ancient protein structural domain, common to vertebrates and many bacterial species.

**Results:**

We show, using bioinformatics approaches, that the P-DUDES domain, surprisingly, adopts the thioredoxin-like (Thx-like) fold. A tentative, more detailed prediction of function is made, namely, that of a 2-Cys peroxiredoxin. Incidentally, consistent overexpression of all three human P-DUDES genes in two public glioblastoma microarray gene expression datasets was discovered. This finding is discussed in the context of the tumor suppressor role that has been ascribed to P-DUDES proteins in several studies. Majority of non-redundant P-DUDES proteins are found in marine metagenome, and among the bacterial species possessing this domain a trend for a higher proportion of aquatic species is observed.

**Conclusions:**

The new protein structural domain, now with a broad enzymatic function predicted, may become a drug target once its detailed molecular mechanism of action is understood in detail.

## Background

One of the challenges of the "post-genomic era" in biology is the elucidation of molecular functions of hundreds of protein-coding genes which are known to be active in disease-related biological processes, but the nature of their role remains elusive. Such proteins, although often advertised as "potential therapeutic targets", are very difficult to exploit as such. Functional prediction of uncharacterised proteins often gains from focusing on sequence regions not easily assigned to known structural domains. Here, we present a novel thioredoxin-like fold protein family.

Initially, the analysis involved three vertebrate proteins, characterised originally by the presence of coiled-coil domain (CCDC80) and sushi and Hyr repeat domains (SRPX and SRPX2). Genes encoding these proteins were identified in various pathological conditions in humans, rodents and, in one case, in chicken. First reports on the *SRPX *gene (a.k.a. DRS, ETX1) initially linked it to X-linked *retinitis pigmentosa*[1,2], and also identified it as a gene downregulated by *v-src*[3]. Furthermore, it has been found that SRPX suppressed *v-src *transformation without effect on cell proliferation[4]. SRPX2, a paralogue of SRPX, was originally discovered as a "sushi-repeat protein upregulated in leukemia" and named accordingly (SRPUL)[5]. Rat CCDC80 (a.k.a. URB, DRO1, SSG1, equarin, CL2) was identified as a protein upregulated by β-estradiol (E2) in rat mammary tissue and associated with carcinogenesis[6]. The CCDC80 (URB) protein was also identified as upregulated in adipose tissue of mice deficient in bombesin receptor subtype-3[7]. The chicken CCDC80 (equarin) was found in eye lens where it is involved in formation of the eye[8].

The similarity of the C-terminal regions of SRPX and SRPX2 proteins, referred to herein as P-DUDES domain, to the three repeat regions in CCDC80 was noticed early on[6,8]. Although the sequence similarity of the P-DUDES domains in SRPX, SRPX2 and CCDC80 has been discussed on many occasions, the biological functions of these proteins were usually not considered together, since no functional features were recognized within the mutually similar sequence regions. In addition, it has been explicitly stated that SRPX and CCDC80 were not functionally related[6]. In our work, we described not only the vertebrate examples of the domain, named by Bommer *et al*., DUDES (DRO1-URB-DRS-Equarin-SRPUL)[9], but also the many prokaryotic homologs. This is why we decided to rename the domain to P-DUDES (Prokaryotes-DUDES).

In recent years, more reports on P-DUDES proteins appeared, bringing the attention to differential expression of *SRPX*, *SRPX2*, and *CCDC80 *genes in various developmental processes and in various tumors (see Table 1). In general, *SRPX2 *was reported as overexpressed in cancer while *SRPX *and *CCDC80 *were identified as downregulated in malignant conditions. The two latter genes were described as tumor suppressors, and some relevant tumor suppression mechanisms were proposed.

**Table 1:** P-DUDES gene expression in cancer and cancer models

gene	direction	tissue	mechanism proposed	reference
*CCDC80*	DOWN	rat epithelial cells neoplastically transformed by β-catenin.		[9]

*CCDC80*	DOWN	thyroid neoplastic cell lines and tissues		[14]

*CCDC80*	DOWN	colon and pancreatic cancer cells.	mediates growth inhibition	[9]

*SRPX*	DOWN	highly malignant human pulmonary neuroendocrine tumors.		[10]

*SRPX*	DOWN	prostate carcinomas.		[89]

*SRPX*	DOWN	colorectal neoplasms.		[90]

*SRPX*	DOWN	lung adenocarcinomas.		[91]

*SRPX*	DOWN	colon adenocarcinomas.		[92]

*SRPX*	DOWN	endothelial cells, myeloma		[93]

*SRPX*	DOWN	stromal fibroblast cells from liver metastases of colorectal cancer		[94]

*SRPX*	NA	SRPX knock-out mouse embryonic fibroblasts	involvement in the maturation process of autophagy	[13]

*SRPX*	NA	malignant tumors including lymphomas, lung adenocarcinomas and hepatomas	Tumors appear in SRPX-knock out mice	[11]

*SRPX*	NA	human cancer cell lines.	apoptosis induction in human cancer cell lines; both the P-DUDES and the sushi regions necessary	[12]

*SRPX2*	NA	endothelial cells	mediator of angiogenesis	[20]

*SRPX2*	NA		ligand for uPAR	[19]

*SRPX2*	UP	gastric cancer	enhancing cellular migration and adhesion through FAK signaling	[16,17]

*SRPX2*	UP	pro-B leukemia cells		[5]

The P-DUDES gene with links to cancer most broadly documented is *SRPX*. It was found to be downregulated in malignant pulmonary neuroendocrine tumors[10] whereas its downregulation was correlated with the malignancy of the tumor (most downregulation observed in tumors is related to shortest survival time). Of note, 30% of *SRPX *knock-out mice developed various tumors: lymphoma, lung cancer, hepatoma, sarcoma[11], while no tumors appeared in the control wild-type mice. The proposed mechanism of tumor suppression by *SRPX *is induction of apoptosis. Ectopic expression of the *SRPX *protein induced apoptosis in human cancer cell lines. Both the P-DUDES and the sushi repeat regions were necessary for apoptosis induction, and *SRPX *activated caspases-12, -9, and caspase-3 [12]. Reintroduction of *SRPX *into lung cancer cell line from *SRPX *knock-out mice led to the suppression of tumor formation, accompanied by enhanced apoptosis[11]. *SRPX*-mediated apoptosis was correlated with the suppression of tumor formation[11].

However, the tumor suppression mechanism mediated by *SRPX *is more complicated than solely that of apoptosis induction, since a recent report shows that this gene is involved in the maturation process of autophagy induced by low serum, as studied in *SRPX *knock-out mouse-derived fibroblast cultures[13]. Thus, P-DUDES proteins may mediate both apoptosis and autophagy which suggests for its broader function.

Similarly to *SRPX*, the *CCDC80 *gene was found to be downregulated in colon and pancreatic cancer cells, and also downregulated in cell lines by multiple oncogenes (β-catenin, γ-catenin, *c-myc*, *h-ras*, *k-ras*, and *GLI*)[9]. Furthermore, *CCDC80 *downregulation was seen in human thyroid neoplastic cell lines and tissues[14]. In a manner somewhat reminiscent of *SRPX, CCDC80 *was found to suppress anchorage independent growth and sensitize cells to anoikis and *CD95*-induced apoptosis. Interestingly, CCDC80 protein is localised to endothelial cells of the vasculature of the tumors[6] which may suggest a role in angiogenesis for CCDC80. Of implication to a role in cancer is the observation that mouse CCDC80 is involved in assembly of extracellular matrix and mediates cell adhesiveness[15].

In contrast to the two other P-DUDES genes (*SRPX *and *CCDC80*) that are almost always downregulated in cancer, the *SRPX2 *gene was reported to be overexpressed in gastric cancer[16], see also Table 1. Of note, prognosis in gastric cancer is related to *SRPX2 *expression (patients with unfavorable prognosis had significantly higher *SRPX2 *expression than those with more favorable one[16]). It has been suggested that *SRPX2 *might be treated as a prognostic biomarker associated with a malignant gastric cancer phenotype[17]. Recent studies have shown that *SRPX2 *overexpression significantly enhanced cellular migration and adhesion[16]. It was also demonstrated that these cellular features caused by *SRPX2 *overexpression are dependent on the focal adhesion kinase (FAK, PTK2) signalling pathway and that *SRPX2 *overexpression increased phosphorylation of FAK at the autophosphorylation site Y397, and at the activation loop site Y596-Y597[16]. The FAK kinase plays a crucial role in tumorigenesis, cancer progression and metastasis[18].

The SRPX2 protein was also discovered to be a ligand for plasminogen activator receptor (UPAR), a key molecule involved in invasive migration of angiogenic endothelium[19]. SRPX2 protein was shown to interact with extracellular domains of UPAR, and to bind the UPAR on cell surfaces[19]. SRPX2 was also found to interact with the protease cathepsin B (CTSB) and the metalloproteinase ADAMTS4 which are components of the extracellular proteolysis system. Thus, SRPX2 may be involved in regulating the proteins involved in the proteolytic remodeling of the extracellular matrix[19]. *SRPX2 *gene was strongly upregulated in angiogenic endothelial cells as compared to resting ones, and silencing the *SRPX2 *delayed angiogenesis[20].

Apart from cancer-related roles, *SRPX2 *is implicated in neurological disorders. Two non-synonymous mutations in *SRPX2 *are known. Y72S, located in the first sushi domain, is associated with mental retardation, rolandic epilepsy and perisylvian polymicrogyria[21]. A gain-of-glycosylation mutation, N327S, located in the P-DUDES domain, close to its N-terminus, is associated with rolandic epilepsy, oral and speech dyspraxia and mental retardation[21]. Thus, the role of SRPX2 in disorders of the speech cortex may involve regulation of proteolytic remodeling of the extracellular matrix[19].

The "clan" of thioredoxin-like (Thx-like) proteins is a large and diverse group sharing the common structural domain, and catalyzing related redox reactions using conserved cysteine residues[22]. Typical reactions for Thx-like proteins include disulfide bond formation and reduction, protein glutathionylation/deglutathionylation and hydroperoxide reduction. Notably, several groups of Thx-like proteins lack one or both cysteines from the archetypic CxxC motif common to most thioredoxin-like molecules.

In this paper, we first present the Thx-like structural prediction for the P-DUDES family. Then, we discuss the relevance of the structure predictions for their predicted molecular function. Furthermore, we analyse the characteristics of the bacterial species possessing the P-DUDES domain proteins. We also summarise the available functional relationship information for the human P-DUDES as well as the available data on cancer-related P-DUDES expression differences, and present evidence on the P-DUDES link to glioblastoma progression.

## Results

### Identification of the P-DUDES domain

The regions of similarity common to the three P-DUDES proteins, as identified by several authors (e.g.[9]), were explored in order to elucidate their potential relationship with sequences of known functions. Since PSI-BLAST searches starting from the human P-DUDES domain sequences yielded significant similarity to known peroxiredoxins structures within 3-7 iterations (see Table 2), we sought to survey P-DUDES and similar proteins in the sequence "hyperspace". To this end, the Saturated BLAST procedure was employed with the C-terminal part of the human SRPX protein [Swiss-Prot:P78539], region 305-464, as query, using the standard parameters[23] (see Methods for details). This procedure, which performs a cascade of PSI-BLAST searches, using representative significant hits as queries in subsequent Saturated BLAST iterations, yielded after seven iterations 4721 "hit" sequences from the nr and env_nr databases. Since such a search can easily "drift out" of the original (P-DUDES) sequence family, the hit sequences were checked for presence of proteins that could be assigned to already known structural domains using the well-established Pfam domain classification system[24] that distinguishes protein domain families, sometimes grouped in "clans". In the total hit sequence population, majority was easily assigned (using HMMER on the Pfam database) to one or more of several Pfam families of the thioredoxin-like clan: AhpC/TSA (PF00578, 4162 sequences), redoxin (PF08534, 2515 sequences), SCO1/SenC (PF08534, 366 sequences), Glutathione peroxidase (PF00255, 11 sequences) and DUF899 (PF05988, 1 sequence). Some proteins were given overlapping assignments to two or more similar Pfam domains. The hit set included ten proteins of known structure, mostly peroxiredoxins. Among the first proteins of known function encountered during the PSI-BLAST searches, *Sulfolobus sulfataricus *bacterioferritin comigratory protein-1 (Bcp1) and *Plasmodium yoelli *Thioredoxin Peroxidase I were found (see Table 2). These proteins belong to the thioredoxin-dependent peroxidase family (AhpC-TSA) and were shown to preserve their function due to the crucial cysteine (Cys45) residue[25]. Out of 4721 "hit" sequences, 559 had no significant Pfam family assignment (HMMER E-value below 0.01). These, after the removal of redundancy at 70% sequence identity threshold, yielded a set of 145 sequences which was treated as a representative set of the P-DUDES domain. Strikingly, out of the 145 sequences, those with assigned organism of origin belonged to two phylogenetic groups: *Bacteria *(58 sequences) and *Vertebrata *(32 sequences). The rest (65 sequences) were assigned to marine metagenome, i.e. environmental samples from Sargasso Sea[26], and thus it cannot be ruled out that other phylogenetic groups, e.g., *Archaea*, possess proteins with a P-DUDES domain. The bacterial P-DUDES proteins included representatives of alpha-, beta- and gamma Proteobacteria, Cyanobacteria, high GC bacteria, and the *Bacteroidetes*/*Chlorobi *group (CFB group) of Eubacteria.

**Table 2:** Significance scores for structure predictions for selected P-DUDES domains

PSI-Blast hits in the PDB database			
**domain**	**First PDB hit (PDB ID)**	**iteration**	**E-value**

CCDC80-II	2h01|A, *Plasmodium yoelii *Thioredoxin Peroxidase I	4	4e-05

CCDC80-I	3drn|A, *Sulfolobus sulfataricus *Bcp1	6	8e-08

CCDC80-III	2h01|A	3	2e-04

SRPX	3drn|A	4	4e-04

SRPX2	3drn|A	3	0.004

**FFAS hits in the PDB database. Z-score below -9.5 means significant similarity (less than 3% of false positives)**[27]			

**domain**	**First PDB hit (PDB ID)**	**Sequence identity**	**Z-score**

CCDC80-II	3drn_A	12	-35.5

CCDC80-I	3drn_A	11	-18.6

CCDC80-III	3drn_A	9	-40.1

SRPX	3drn_A	10	-30.3

SRPX2	3drn_A	15	-35.7

**HHpred hits in the PDB database**			

**domain**	**First PDB hit (PDB ID)**	**E-value**	

CCDC80-II	3drn_A	0.00093	

CCDC80-I	3drn_A	0.00081	

CCDC80-III	2h01_A	1.1E-20	

SRPX	3drn_A	0.0011	

SRPX2	3drn_A	0.00018	

In order to ascertain fold prediction for the P-DUDES domain, the FFAS03[27] method was employed, yielding significant and consistent prediction of thioredoxin-like superfamily (c.47.1) in the SCOP database (see Table 2). Also, HHPred[28] decidedly ascribes the P-DUDES family to the same SCOP superfamily (see Table 2). More specifically, the FFAS03 and HHPred structure prediction methods consistently flagged the peroxiredoxin (AhpC/TSA) family proteins as the closest structural matches for the P-DUDES domain. It has to be borne in mind that structural predictions are not automatically extendable to function predictions. Thus, functional meaning of the structural assignment for P-DUDES proteins will be discussed further down.

**Figure 1:**
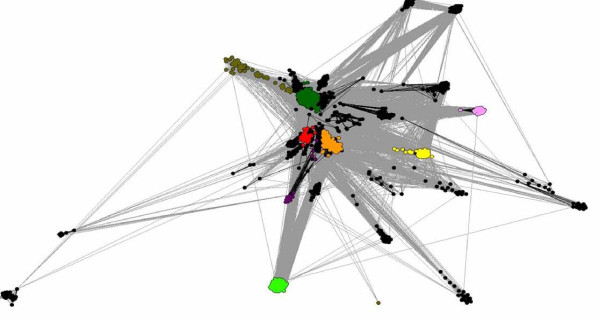
**Diagram (Clans) showing relationships between P-DUDES proteins and 27 different Pfam families of the Thioredoxin-like clan**. Dots (nodes) represent sequences, and lines (edges) represent significant PSI-BLAST similarities. Light green: P-DUDES, yellow: TrbC, pink: DUF2703, light blue: ArsD, orange: AhpC/TSA and Redoxin, magenta:DUF929, red: Thioredoxins, dark green: Glutaredoxin, khaki: GST_N, dark blue: DUF3088

Recently, Atkinson and Babbitt[22], presented a thorough survey of the thioredoxin-like clan members using a graph representation of pairwise sequence similarities. Here, their Thx census is being extended into predicted novel member families. The relationship of the P-DUDES family to the Thx clan was visualized using a similar approach, the CLANS algorithm[29]. The Clans graph visualizes PSI-BLAST-detected significant similarities. Notably, P-DUDES appears as a bona fide "fringe" member of the clan, with strong links to central families (Redoxin, AhpC/TSA), but also a number of other families (19 out of 36) - see Figure 1. Incidentally, examination of distant FFAS sequence assignments for P-DUDES allowed us to classify several other Pfam domains (DUF929, DUF1094, DUF1462, DUF2847, DUF1223, ArsD, DUF2703, DUF3088 and TrbC_Ftype) as members of the thioredoxin-like clan not recognized as such in the recent Pfam 24.0 database release (see Table 3). Four out of these families have not, to our knowledge, been identified as thioredoxin-like before (DUF929, DUF2703, DUF3088 and TrbC_Ftype). The placement of these "novel Thx" domains in the CLANS graph is relatively central. Most of these are families of single-domain proteins of unknown function found in Bacteria or Archaea. DUF1223 is also found in fungi and plants. ArsD is present in bacterial "Arsenical resistance operon trans-acting repressors", that act as arsenium metallochaperones[30]. Interestingly, a related detoxification system uses another Thx-like domain, ArsC, an arsenate reductase that uses reduced glutathione[31]. TrbC_Ftype domain is found in bacterial "Type-F conjugative transfer system pilin assembly proteins"[32]. DUF2703 is found fused together with two other related domains (ArsD and MOCO sulfurase C-terminal) that support its oxidoreductase function predicted herein. The new Thx-like domains were assigned by significant or borderline, but consistent, HHPred and FFAS predictions, and some were also supported by structure comparison for recently solved structures (see Table 3).

**Table 3:** Similarity between known thioredoxins and the novel Thx-fold families

Pfam domain	Top PDB hit (PDB ID)	p-value	Top Scop hit	p-value	Top Pfam hit	p-value	Comment
ArsD	3ktb_A Arsenical resistance operon trans-acting repressor	0	d1iloa c.47.1.1	8.7E-10	pfam00462 Glutaredoxin	9.2E-07	Structure known, 3ktb, has Thx fold, as seen by Fatcat and Vast

DUF929	1fo5_A Thioredoxin	0.00015	d1fo5a c.47.1.1	0.00021	PF00085 Thioredoxin	0.00026	

DUF1094	3fhk_A YphP disulfide isomerase	0	d1f9ma c.47.1.1	8.8E-06	pfam09085 Adhes-Ig_like pfam00085 Thioredoxin	1.3E-05 0.00035	Structure known, 3fhk, has Thx fold as stated by the authors

DUF1223	2axo_A Hypothetical protein	0	d2axoa1 c.47.1.19	0	pfam00462 Glutaredoxin	7.5E-08	Structure known, 2axo, classified as Thx in Scop

DUF1462	1xg8_A Hypothetical protein	0	d1xg8a c.47.1.17	0	pfam05768 DUF836 Glutaredoxin-like	1.5E-05	Structure known, 1xg8, classified as Thx in Scop

DUF2703	1ilo_A Conserved hypothetical protein	8.6E-06	d1iloa c.47.1.1	1.7E-05	PF03135 CagE_TrbE_VirB pfam00462 Glutaredoxin	0.00024 0.00061	

DUF2847	3iv4_A Putative oxidoreductase	0	d1ep7a c.47.1.1	1.8E-10	pfam00085 Thioredoxin	2.1E-08	Structure known, 3iv4, has Thx fold, as stated by the authors

DUF3088	3ir4_A Glutaredoxin 2	1.6E-06	d1k0ma2 c.47.1.5	1.6E-06	PF02798 GST_N	0.00012	

TrbC_Ftype	1dby_A Chloroplast thioredoxin	6.7E-07	d1dbya c.47.1.1	5.8E-07	PF00085 Thioredoxin	4.1E-06	

Similarity scores (HHPred) supporting similarity between thioredoxins

and the nine novel Thx-fold families ArsD, DUF929, DUF1094, DUF1223, DUF1462, DUF2703, DUF2847, DUF3088 and TrbC_Ftype.

### Domain structure of the P-DUDES proteins

**Figure 2:**
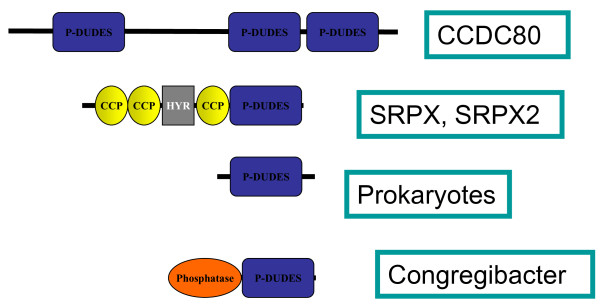
Diagram showing domain composition of representative P-DUDES proteins

The vertebrate P-DUDES proteins are multidomain, possessing one (SRPX and SRPX2) or three (CCDC80) P-DUDES domains (see Figure 2). The three genes appear to be conserved in all vertebrates for which full genome information is available, although in fishes (e.g. *Danio rerio, Tetraodon nigroviridis*) there are three distinct paralogues of the *CCDC80 *gene. The similarities of vertebrate P-DUDES domains (see the phylogenetic tree in [Additional file 1: Supplementary Fig. S1]) suggest that a), the common ancestor of vertebrates had a duplicated *SRPX*-like gene (all vertebrates analysed have orthologues of both human *SRPX *and *SRPX2*) and b) the common ancestor of vertebrates had at least one *CCDC80*-like gene. P-DUDES domains appear to be absent from non-vertebrate chordates, and all other metazoans.

The SRPX and SRPX2 proteins possess three CCP (complement control protein) modules (also known as short consensus repeats SCRs or sushi repeats, Pfam identifier PF00084), each approximately 60 amino acid residues long[33]. Sushi repeats are common in two groups of proteins. In selectins, membrane-bound proteins involved in inflammation, sushi are extracellular and most probably take part in mediation of blood cells movement on inflammation-activated endothelium[34]. Sushi repeats are also abundant in various complement system proteins[35]. In between the second and the third sushi module in SRPX, the hyalin repeat (Hyr, Pfam identifier PF02494) can be found which is supposed to be involved in cell adhesion[36]. The arrangement of several sushi repeats seen in SRPX and SRPX2, is also present in selectins. Similarity between SRPX-like proteins and selectins is relatively high, for example, human SRPX and E-selectin share 25% identical residues over 269 positions. The co-occurrence of sushi and Hyr domains suggests that SRPX proteins are located extracellularly. This is in accordance with the experimental data on extracellular expression of the P-DUDES proteins (see the Introduction section).

The search for short, nearly exact sequence matches using the threonine-rich low-complexity region of chicken CCDC80 (equarin, residues 328-395) as query revealed the similarity to a secreted protein (low density lipoprotein receptor-related protein 8). This suggests the involvement of this sequence in extracellular matrix attachment. The CCDC80 lysine-rich region (residues 495-610) is predicted to be a coiled-coil structure[37]. More broadly, the central region of human CCDC80, approximately the residues 300 to 600, between the first and second P-DUDES domains is predicted to be disordered[38]. Thus, in CCDC80, between P-DUDES domain 1, 2 and 3 there seems to be a large, flexible or disordered linker domain that may allow extensive interactions between the covalently linked domains.

The non-vertebrate P-DUDES proteins (bacterial and metagenomic ones) are typically shorter, single domain molecules, with just one exception (see Fig 2). In *Congregibacter litoralis *KT71 protein [GenBank:88706344], a P-DUDES domain is found at its C-terminus, together with a calcineurin-like phosphoesterase domain (Pfam Metallophos, PF00149 [39]). A similar arrangement involving an AhpC-TSA (peroxiredoxin) domain together with a calcineurin-like phosphoesterase domain is found in the bacterium *Chitinophaga pinensis *DSM 2588 protein, [GenBank:256420021]. Such an arrangement of two enzymatic domains, although not studied experimentally, suggests the possibility of mutual regulation of the two enzymes.

Out of the 145 representative P-DUDES domain sequences, vast majority (including all vertebrate proteins) were predicted to be extracellular (TMHMM program). Approximately half of the proteins (again including all vertebrate ones) had signal peptides as predicted by the SignalP algorithm[40].

### Structure models of the P-DUDES domain

**Figure 3:**
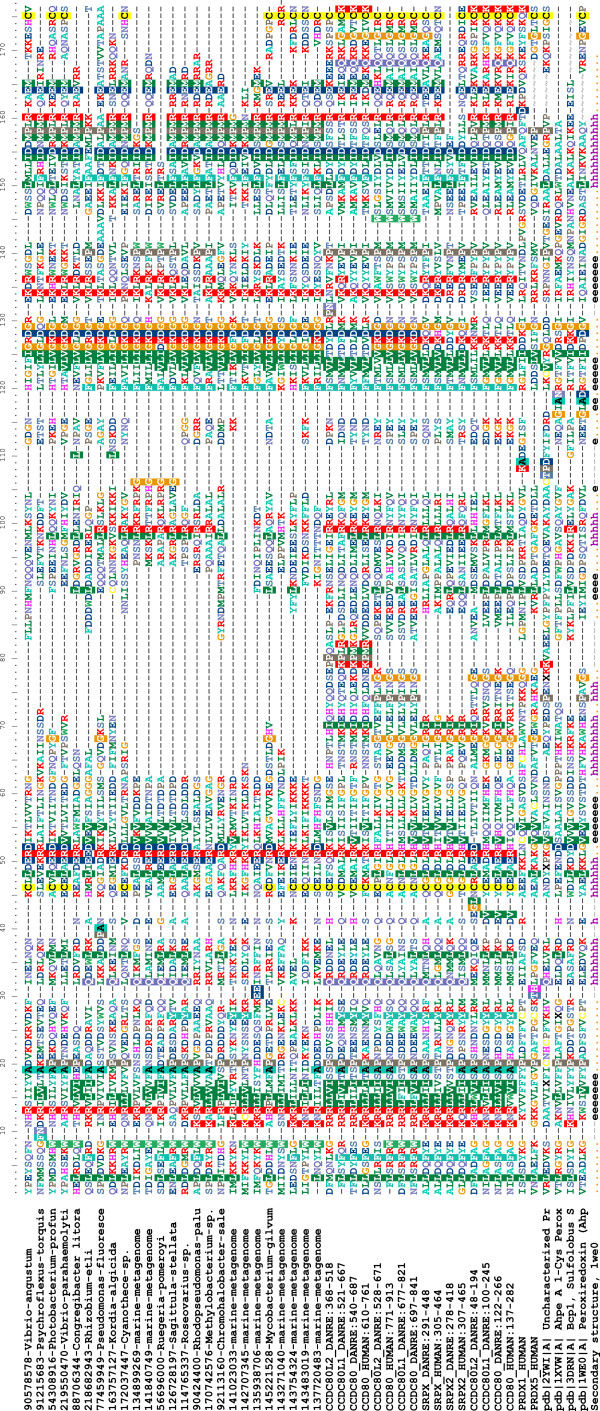
**Sequence alignment (Muscle) of selected P-DUDES domains together with selected known peroxiredoxins added, among them the templates used in structure modeling**. Secondary structure, as determined in the 1we0 structure, indicated. For a complete version of this alignment, see [Additional file: Suppl. Fig. S2].

Secondary structure predictions, and sequence alignments to known Thx-like structures produced by structure prediction methods show that the P-DUDES domains are composed of four beta strands forming a central beta sheet, and three or four alpha helices. In terms of the thioredoxin fold nomenclature of Atkinson and Babbitt[22], which is a modified version of that of Qi and Grishin[41], P-DUDES domain secondary structure order is as follows: β-1, α-1, β-2, α-2, β-3, β-4, α-3. These secondary structure elements form the core of a typical thioredoxin-like fold protein[41]. Many bacterial and metagenomic P-DUDES sequences lack most or all of the helix α-2, which is also absent in some Thx-like structures. Also, vertebrate P-DUDES proteins differ in the length of the α-2 region, with SRPX and SRPX2 having the shortest, and CCDC80 domain 3 having the longest α-2 region. The Thx-like fold proteins are known to vary in their structures around the typical "classic" arrangement, and exhibit even cases of circular permutations of secondary structure elements, where a region from the N-terminus of a protein may be "moved" to the C-terminus or vice versa[41]. As seen in the multiple sequence alignment in Figure 3, the central region of the alignment (columns 60-110) is of variable length and poorly alignable. In the typical peroxiredoxin template used herein for P-DUDES structure modeling (1we0), this region includes the helix alpha-3, an "extra" beta strand that extends the central beta sheet, and a short "extra" alpha helix. This whole stretch of the polypeptide chain forms a large "loop" on the periphery of the central fold (mainchain of residues 73 and 116 approach each other to within 6 Å). In P-DUDES domains of SRPX/SRPX2 and all microbial sequences, this region is short (approx. 10-20 residues) and in some marine metagenomic P-DUDES proteins it is missing altogether. Thus, the poorly alignable region of P-DUDES can be seen as "dispensable" to the structure. In support, for the vertebrate P-DUDES sequences, the central sequence region obtains highest scores for the disordered state prediction (data not shown).

From among the many possible structure modeling templates, three were selected: "Uncharacterized Conserved Protein", *Geobacillus kaustophilus *[PDB:2ywi], "AhpE, a 1-Cys Peroxiredoxin", *Mycobacterium tuberculosis*, [PDB:1xvw] and "Peroxiredoxin (Ahpc)", *Amphibacillus xylanus*, [PDB:1we0]. These templates had both favorable FFAS score for sequence comparison with the P-DUDES domains, indicating reliable structural similarity, and possessed a cysteine residue at the C-terminus of the peroxiredoxin domain, which serves the function of a "resolving cysteine" in the peroxiredoxin catalytic mechanism[42]. Since the cysteine at the C-terminus is a characteristic feature of the P-DUDES domain (also see discussion further below), it was supposed that it may be a factor in the catalytic mechanism. Also, the templates representing both types of peroxiredoxin dimer interfaces were chosen (see below). The three modelling templates belong to the AhpC/TSA Pfam family. A series of 15 structure models were built, including models of all five human P-DUDES domains, multiplied by the three templates chosen.

Since, most known peroxiredoxins function as homodimers or higher order multimers of homodimers[43], P-DUDES domain structural models were built as such homodimers. Two basic dimeric arrangements are known for peroxiredoxins, so called "A-type" and "B-type" interface forms[43]. Two of the templates exhibited "type-A" interface, whilst the third one (1we0) - the "type-B" one. In the "B-type" interface, the beta-4 strands of the two monomers form an "edge to edge" association, making up an extended 8-stranded beta-sheet. In the "A-type" interface, the dimer interface is made-up mostly by residues from helices alpha-1 and alpha-3. However, model quality parameters (Modeller and MetaMQAP, see Methods section) were not sufficient to select a preferred model out of the alternative P-DUDES dimer models.

Although we present structure models of P-DUDES domains in dimeric arrangements as speculative ones, comparing the alternative dimeric arrangements, for the SRPX2 protein Western blot data suggest the presence of a dimer alongside with the monomer in the extracellular medium[16].

### The relevance of the structure predictions for molecular function

The sequence similarity of P-DUDES to peroxiredoxins and to bacterial comigratory proteins (BCPs), in particular, may reflect only the general fold similarity because the cysteine residue, important for peroxiredoxin function is not conserved in the P-DUDES domain. However, 53 out of 145 analysed P-DUDES domains, including all vertebrate ones, possess a conserved cysteine residue, present also in *Mycobacterium*, *Cyanothece*, all the analysed *Vibrionales*, and a number of metagenomic sequences (see Figure 3 and [Additional file 1: Suppl. Fig. S2]). Although this cysteine is shifted by 12 and 15 residues, relative to the two cysteines in the classic thioredoxin-like active site motif CxxC, we hypothesize that the P-DUDES conserved cysteine may be the peroxidatic residue (CysP). Also, a second cysteine, located at the C-terminus of the P-DUDES domain is strictly co-conserved with the putative peroxidatic one (see Figure 3). The Fisher's exact test *p*-value, which estimates the significance of the co-occurrence of the two cysteine residues, is less than 10^-10^. Thus, we hypothesize that the C-terminal cysteine in P-DUDES may serve the role of the resolving cysteine (CysR)[42]. This residue is used in "typical 2-Cys peroxiredoxins" to restore the starting state of CysP[44].

The putative peroxidatic cysteine of the P-DUDES domain is located within a C-x(4)-R motif (see logo in [Additional file 1: Suppl. Fig. S3]), whereby the Arg residue is even more conserved than the cysteine itself. As discussed by Poole and colleagues, an arginine close to the peroxidatic cysteine may stabilize the active site thiolate anion[42]. However, distance between the Arg Nε and the Cys Sγ atoms in our models of P-DUDES domains is on the order of 16 Å (Cα-Cα distance is on the order of 10 Å), which makes a direct interaction between Arg and Cys-bound substrate less likely.

Hence, the function prediction of peroxiredoxins for P-DUDES domains is supported by a) the significant sequence similarity to peroxiredoxins, and b) the frequent presence of a conserved pair of Cys residues. However, this prediction is speculative. The first conserved P-DUDES Cys residue is located at a different location than the "classic" peroxidatic cysteine of peroxiredoxins. The former is in the C-terminal part of helix α-1 or in a loop between α-1 and the strand β-2, while the latter - at N-terminus of helix α-1 or in a loop between the strand β-1 and the helix α-1[44]. Thus, the putative catalytic cysteine of P-DUDES domain is exposed on a different "face" of the molecule than the typical thioredoxin active site cysteine.

Also, the monomer surface in the vicinity of CysP in the structure models of P-DUDES is rather convex (see [Additional file 1: Suppl. Fig. S4]), not allowing for a typical substrate-binding cleft. Yet, a cleft formed by a rearranged C-terminus of the P-DUDES molecule or by the other P-DUDES chain in a dimer might be plausible. Moreover, the structure models built using templates identified by remote sequence similarity relationships (in this case, 9-15% identical residues over 140 positions) are of an illustrative rather than predictive nature, and the fine features of the molecular surface cannot be treated with certainty[45].

The CxxxxR motif does exhibit some conserved features, it can be summarised more precisely as a regular expression Cx[LIFM][DE][DE]R, where square brackets indicate alternative residues at a position. As observed in P-DUDES models, the sidechains of Asp or Glu residue at position 5 in the motif can stabilize the Cys residue by a hydrogen bond to the cysteine SH group, and itself be stabilized by a salt bridge to the Arg residue at position 6 in the motif.

The molecular surface near the presumed CysP catalytic residue in human P-DUDES proteins is not formed of strictly conserved residues, and its electrostatic potential or lipophilic "potential"[46] are not conserved (see [Additional file 1: Suppl. Fig. S4]). Some hydrophobic surface segments are formed near CysP by Leu residues at position 5 in the CxxxxR motif or by aromatic Phe/Tyr residues preceding the CysP. In addition, some areas of negative potential are formed by the acidic residues at positions 2 or 5 in CxxxxR. However, even in the small group of five human P-DUDES domains, these features are not conserved. In contrast to P-DUDES, at the active site of peroxiredoxin from *Aeropyrum pernix *K1, with peroxide H_2_O_2 _bound[47], the surface near the CysP has a positive electrostatic potential, which is brought by an Arg residue distal in sequence. The peroxide molecule interacts with CysP, and with backbone amine groups, as well as with sidechain of a Thr residue located 3 positions before CysP.

Furthermore, for the second conserved P-DUDES cysteine residue, the distance to the presumed catalytic cysteine is prohibitive for direct interaction (on the order of 25Å), but a structural rearrangement can be imagined, including either the C-terminal helix and tail, or the corresponding region from the other chain.

Variable location of the active site peroxidatic cysteine in thioredoxin fold proteins has been noted before[48], however this variation involved shifts of a few residues close to the N-terminus of helix α-1. In the P-DUDES family, besides the "typical P-DUDES cysteine location" at the C-terminus of helix α-1, several alternative cysteine locations are found, allowing a hypothesis that other ways of arranging active sites are also possible in the P-DUDES structural framework, and also providing speculative "intermediate" solutions, between the "classic Thx" and "classic P-DUDES" locations. These include Cys locations in the loop β-1/α-1, at the N-terminus of helix α-1, at the N-terminus of strand β-1, and within the strand β-2. All these alternative Cys locations are found in proteins from marine metagenomes lacking the CxxxxR motif. An example of a thioredoxin fold proteins, where the active site has been "shifted" away from the canonical CxxC location at the N-terminus of helix α-1, are glutathione transferases[49-51] in which the active site residue varies between tyrosine, serine and cysteine[52,48]. In the recent exhaustive survey of thioredoxins-like fold proteins, Atkinson and Babbitt[22] noted that 22% of Thx-fold sequences had none of the two archetypical catalytic Cys residues conserved. Most of these belonged to the glutathione transferase (GST_N) family.

In summary, the hypothesis that the CxxxxR motif in P-DUDES is responsible for the peroxiredoxin function is far from being proven. Two alternatives can be envisaged. Firstly, P-DUDES proteins may not serve the oxidoreductase function, in contrast to the overwhelming majority of the Thx-like fold proteins. Secondly, oxidoreductase function in P-DUDES may be mediated by residues other than the CxxxxR motif, for example the [DN]xx[YF] motif that aligns perfectly with the CxxC active site motif of thioredoxins in HHPred analysis. This may be plausible, since in tyrosine-type glutathione transferases, the catalytic residue is a tyrosine, although the mechanism of its interaction with glutathione is not fully understood[48].

### Conserved sequence motifs and dimeric structure

**Figure 4:**
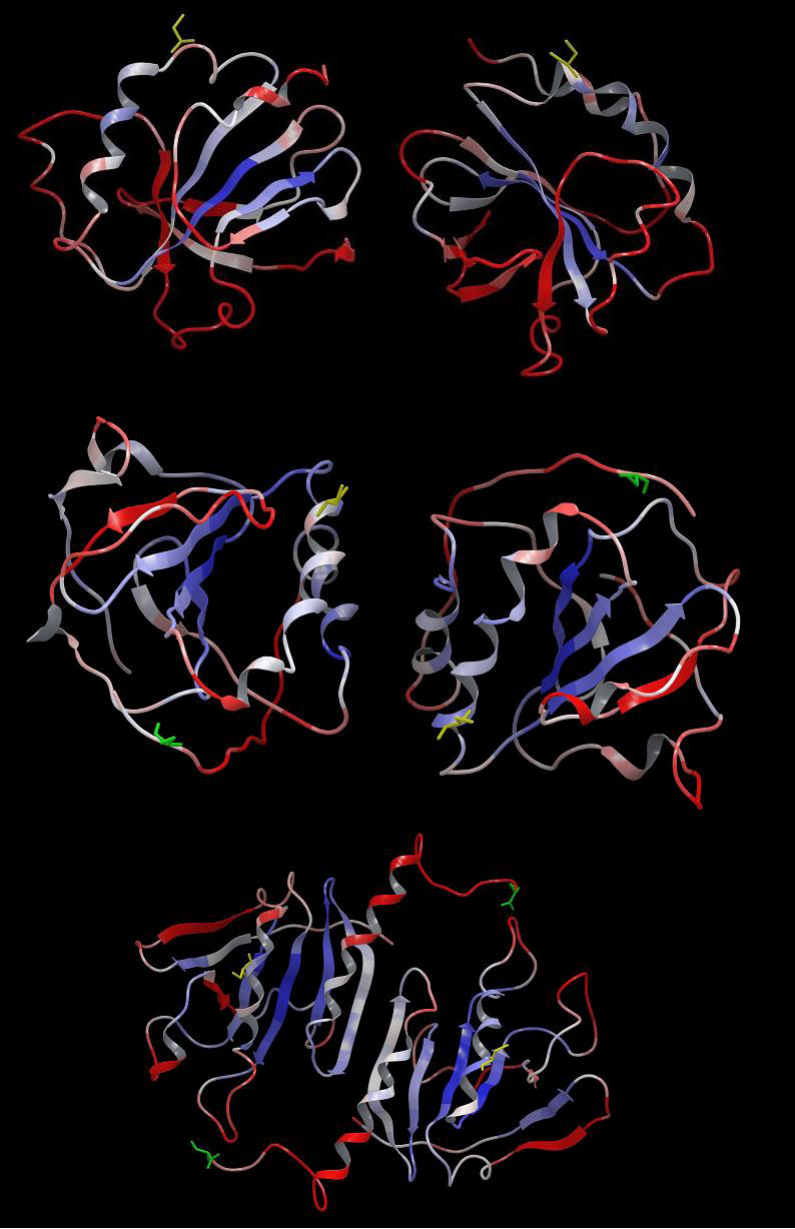
**Structure models of P-DUDES domains from human SRPX in dimeric arrangement**. Colouring is by MetaMQAP score that reflects predicted model accuracy: blue - high accuracy, red: low accuracy. Putative Cys-P (yellow) and Cys-R (green) residues shown. Models were built using the following templates: 1xvw (top), 2ywi (center), 1we0 (bottom). The latter is a "B-type" dimer, the others are "A-type" dimers.

The proteins of the P-DUDES family exhibit similarity to the atypical 2-Cys peroxiredoxins, whereas most often the active unit is a dimer, and the CysP-CysR interaction is of intermolecular type. This is, most likely, also the case for P-DUDES domains, since in the model of a typical P-DUDES domain the distance between the Sγ atoms of CysP and CysR is on the order of 25 Å (see Figure 4). The intermolecular distances between CysP and CysR in alternative dimer models are also large (more than 25 Å); however, the models are not accurate, and peroxiredoxins are known to undergo substantial conformational rearrangements upon transitions between the reduced and oxidised forms. It is noteworthy that all three P-DUDES domains in CCDC80 proteins have retained their putative catalytic Cys residues. A hypothesis could be that they function as a functional "hetero-trimer" of subunits, possibly as a part of a larger multimer. This would be remotely reminiscent of a "pentamer of dimers" arrangement seen for most bacterial peroxiredoxins[43].

Besides the CxxxxR motif (residues 367-372 in human SRPX sequence numbering) and a putative resolving cysteine at the C-terminus, other conserved P-DUDES sequence features include: arginine at the N-terminus of strand β-1 (R34), an aspartate and a lysine between strands β-3 and β-4 (K426, usually within a GxDGxxK motif, res. 420-426), and an IDxxxxRxxE motif (res. 442-451), often more specifically IDxMPMRxxE, in the region covering helix α-3 (see Figure 3 and sequence logos in [Additional file 1: Suppl. Fig. S3]).

The plausibility of three different modeling templates could be judged by the extent of the inter-chain interface, the presence of conserved motifs on the interface, and the physico-chemical features of the surface (see [Additional file 1: Suppl. Fig. S5, Suppl. Fig. S6])*. *In the case of P-DUDES proteins, the inter-chain interface does not differ significantly between the alternative dimeric arrangements. The B-type dimer interface (template 1we0), that has the β-4 strands of the two chains paired, thus forming an intermolecular eight-stranded sheet, buries 1800-2400 Å^2 ^of protein surface in models of various human P-DUDES domains. The other A-type dimer interface (template 2ywi), formed mostly by helices α-1 and α-3, buries 800-1600 Å^2^.

Consurf analysis of sequence conservation mapped on protein surface (see [Additional file 1: Suppl. Fig. S7]) shows that the evolutionarily most conserved surface features in the dimer interface of the SRPX/2ywi model (model of SRPX built on the 2ywi template) are L432 of strand β-4, R372 of the CxxxxR motif in the α-1/β-2, and D443 and R448 of the IDxxxxRxxE motif in helix α-3. The latter motif contributes a conserved inter-chain salt bridge R448-E451. Conserved surface features in the interface of the SRPX/1we0 model are limited to K426, R428 and L432, both in the strand β-4, and R448 in helix α-3, and the conserved inter-chain hydrophobic interaction F445-L447.

In the examination of the physicochemical properties of alternative dimer interfaces (see [Additional file 1: Suppl. Fig. S5]) no consistent electrostatic potential pattern could be observed for the five human P-DUDES domains in either dimer type model (A or B). The lipophilic "potential" maps for the two interfaces consistently shows hydrophobic patches, originating mostly from helix α-3, with more lipophilic character seen in the B-type interface (models on 1we0 template).

In summary, judging from interface size, presence of conserved residues on the interface surface, and physical features of the surface, the B-type interface seems to be slightly more probable. However, this remains to be confirmed experimentally. Moreover, although dimer is the prevalent form among known peroxiredoxin structures, it cannot be ruled out that P-DUDES domains function as monomers or different multimers.

### Bacterial P-DUDES domain proteins

**Figure 5:**
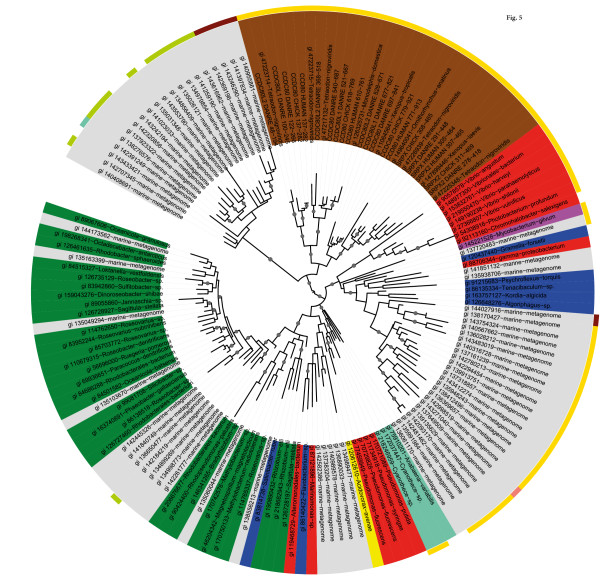
**Phylogenetic tree (PhyML) of 145 representative P-DUDES domains**. Coloured according to taxonomic group of origin (inner pie chart, brown: vertebrate, grey: marine metagenome, red: gamma-proteobacteria, pink: high-GC group bacteria, blue: CFB group bacteria, turquoise: Cyanobacteria, yellow: beta-proteobacteria, green: alpha-proteobacteria) and presence of the presumed active site cysteine residues (outer ring, yellow : "typical" P-DUDES (Cys-P, Cys-R) arrangement, brown: ICK motif instead of CysP, green: KCR motif, pink: KCV motif, aquamarine: TCY motif). Branches with support values > 0.8 are marked with grey circles.

The spread of P-DUDES domains into evolutionarily distant bacterial taxa precludes a definite statement regarding the origin of the group (see Figure 5). Interestingly, although majority of the marine metagenomic P-DUDES sequences do group with alpha-Proteobacteria, gamma-Proteobacteria, and the CFB group bacteria, a substantial cluster of marine metagenome P-DUDES sequences group together with vertebrate domains (Figure 5). It would be tempting to speculate about the taxonomic identity of the latter metagenomic cluster. A similar picture is obtained when the tree is constructed using a different multiple alignment method, Promals vs Muscle, and a different phylogenetic tree construction algorithm, BioNJ vs PhyML (data not shown). Overall, the phylogenetic tree of the representative P-DUDES domains does group the sequences accordingly to their taxonomic assignment. Such a phylogenetic distribution of P-DUDES genes may reflect several alternative evolutionary histories (see Discussion section).

The P-DUDES-possessing bacteria were checked for habitat and lifestyle preferences. No obvious trends were observed for the whole P-DUDES group or for the subgroup of P-DUDES bacteria with the presumed peroxidatic Cys residue conserved. Yet, the "Cys subgroup" had a higher proportion of aquatic species than the rest. Most P-DUDES species were mesophilic, and a minority was pathogenic with a diverse host range including humans, insects and corals. The only significant difference between the Cys subgroup and the rest of P-DUDES bacteria was oxygen requirement, "Facultative" and "Aerobic", respectively (Fisher's Exact Test *p*-value = 0.008).

STRING[53] analysis did not show any co-expression conservation. Likewise, analysis of genomic environments of the P-DUDES bacterial species did not yield strong clues as to the functional roles of the P-DUDES family.

### Evidence for the P-DUDES link to glioblastoma progression

In many cancer-related microarray experiments (see Introduction section), P-DUDES genes showed diverse expression changes, although most often *SRPX *and *CCD80 *were downregulated whereas *SRPX2 *was upregulated. Because of the peroxiredoxin functional hypothesis and cancer-related changes observed, we speculated that P-DUDES proteins might be involved in a common biological mechanism. Furthermore, we speculated that common function might be detectable in an environment or situation where all three genes would exhibit similar behaviour. Thus, we used the Gene Chaser tool[54] to search for microarray experiments where all three human P-DUDES genes would show similar expression changes. The only disease-related datasets where all the three P-DUDES genes showed significant and consistent expression changes (as determined by *q*-values in GeneChaser) were two glioblastoma expression datasets GDS1813 and GDS1962[55,56]. Comparisons considered here involved samples classified as "normal vs glioblastoma" and "non-tumor vs glioblastomas" in the datasets GDS1813 and GDS1962, respectively. The fold changes in the two experiments were: 4.6 and 5.6 for *SRPX*, 10.6 and 2.5 for *SRPX2*, 10.2 and 5.5 for *CCDC80*, with Student *t*-test *p*-values between 0.005 and 1.5E-08 (See [Additional file 1: Suppl. Fig. S7]). Recently, Park and colleagues[57] offered a meta-analysis of four diverse glioblastoma microarray studies. *SRPX2 *is found among their top regulated genes, (*q*-value 0.0069, average fold change glioma vs normal: 2.4).

Epithelial-to-mesenchymal transition (EMT) has recently gained attention as one of the hallmarks of the transition from localized tumors to metastasing malignancies, whereas the loss of attachment, loss of cell polarity, increased migratory and invasive properties are important features[58]^,^[59]. Recently, Phillips and colleagues[60], analysed subclasses of glioblastomas and found that a tumor sample set termed "mesenchymal subset" had high expression of genes negatively correlated to patient survival. This was one of a number of studies which argued that in glioblastoma, expression profiles predict survival better than histological classifications[61]. Our analysis shows that *SRPX *and *SRPX2 *differ in expression between the mesenchymal subset and the rest of glioblastoma samples (subsets as defined by Phillips et al.[60], fold changes 1.7 and 2.4, respectively, *t*-test *p*-values 5.5E-4 and 1.5E-7, respectively). Incidentally, the Phillips paper[60], points out the similarity of the mesenchymal glioma subset to a number of tissues (bone, synovium, smooth muscle, endothelium, dendritic cells). These are the same tissues that normally express P-DUDES genes (as seen in the Symatlas database, data not shown) [62].

Recently, Iavarone and co-workers[63] elucidated transcriptional network of genes involved in mesenchymal transformation of brain tumors. They computed a "worst prognosis group" of genes, and found that it displayed "mesenchymal features". This set included *SRPX2*, as significantly linked to poor glioblastoma prognosis (*t*-test *p*-value 0.024).

A putative signalling pathway linking *SRPX2 *to the cancer regulator kinase FAK may be elucidated using Ingenuity IPA tools. Since overexpression of *SRPX2 *was shown to lead to an increase in FAK phosphorylation[16], IPA relationship database was queried for kinases that had direct interactions with both FAK (PTK2) and any of known SRPX2 interactors (PLAUR, CTSB and ADAMTS4). The five kinases thus found: LYN, FYN, FGR, PDGFRB, EGFR can be candidates for mediators of SRPX2-regulated FAK phosphorylation. Two of these (PDGFRB and EGFR) are implicated in glioblastoma [64].

Altogether, the various expression studies discussed and analysed here, support the role of P-DUDES genes and proteins in cancer, and specifically link it to glioblastoma. More specifically, we postulate here that P-DUDES proteins are involved in the EMT and may be involved in regulation of the interactions between the cell and the extracellular matrix.

## Discussion

We have demonstrated that the P-DUDES family is a large group of vertebrate and bacterial proteins. We have predicted a thioredoxin-like fold for these proteins, and described the possible specific function of a peroxiredoxin. We noted that despite similarity to 2-Cys peroxiredoxins, a different active site arrangement has to be present for the enzymatic activity. Although found in many bacterial species, a P-DUDES domain containing gene is probably not an "essential" gene, since it is often missing from a species within a genus possessing P-DUDES. For example, it is present in several *Pseudomonas *species, but absent from *Pseudomonas aeruginosa*. Likewise, P-DUDES is present in *Mycobacterium gilvum*, but absent from *Mycobacterium tuberculosis. *Yet, some alpha-proteobacterial genomes (*Roseovarius *sp. HTCC2601 and *Sagittula stellata E-37*) harbour two P-DUDES genes sharing as little as 33% and 36% protein sequence identity, respectively.

Adding to P-DUDES involvement in cancer reported by many authors, with opposite expression changes cited for different genes and different pathologies, we discovered its involvement in glioblastoma, and specifically the link to epithelial-to-mesenchymal transition. This is consistent with a role in regulating the interactions of the cell with the extracellular matrix that is one of conclusions of our systems biology analysis of expression data, and has been reported previously.

It has been previously discussed [44] that peroxiredoxins serve two main functions - one in stress defense, and another in physiological peroxide signalling that involves an array of enzymes of various specificities and sensitivities. It is tempting to speculate that P-DUDES domains provide those "yet unidentified peroxide sensors"[65] and possibly couple peroxide or similar substrate turnover to interactions with other effector proteins that are carried out employing the non-enzymatic domains of P-DUDES proteins (Sushi, Hyr, coiled-coil).

In eukaryotes, reactive oxygen species is not only defended against as a toxic threat, but hydrogen peroxide is also a signalling molecule. Many proteins, enzymes, channels, etc., are redox-regulated[66]. The enzyme Peroxiredoxin 3 (PRDX3) regulates apoptosis signalling by mitochondria[67]. Another precedent for possible P-DUDES involvement in cancer progression is the human peroxiredoxin 1(PRDX1) that controls neuronal differentiation by thiol-redox-dependent activation of the GDE2 protein[68].

Interestingly, Szepetowski and co-workers have recently identified SRPX2 as one of the putative interactors of ERP44 (TXNDC4)[19]. ERP44 is a protein containing three thioredoxin domains, residing in the endoplasmic reticulum. The role of ERP44 is that of a protein disulphide isomerase involved in oxidative protein folding and assembly within the secretory pathway[69]. In the context of well-known, functionally-relevant multimerisation of peroxiredoxins, interaction of a predicted peroxiredoxin domain protein, SRPX2, with an established oxidoreductase of similar fold, ERP44, may suggest involvement of SRPX2 in redox-dependent regulation of the secretory pathway.

The discovery of P-DUDES domains has an interesting evolutionary implication. The presence of rather few P-DUDES genes scattered in the space of sequenced prokaryotic and eukaryotic genomes suggests that it has been the subject of the horizontal gene transfer (HGT). An intuitive conclusion would be that the bacterial P-DUDES genes were ancestral, most probably originating from alpha-Proteobacteria. This hypothesis is supported by the relative commonness of P-DUDES genes in this clade, and also by the presence of pairs of P-DUDES genes exhibiting low sequence identity in some alpha-proteobacterial genomes. Such gene pairs, not observed in other bacterial phyla, suggest ancient gene duplication events. Subsequently, after the spread of P-DUDES genes to other bacterial phyla by Mendelian inheritance and/or HGT, at some point they were introduced to the common ancestor of the vertebrates. The internal fusion events found in eukaryotic genomes would be a natural consequence of such an evolutionary path. Another point that may reinforce such a phylogeny is related to ecological niches in which P-DUDES microbes live and specifically the fact that vast majority of P-DUDES genes come from marine metagenomes and marine bacteria. HGT from marine bacteria to marine facultative pathogens and later to vertebrates seems to be a possible way of inheritance of P-DUDES by vertebrates. However, such an evolutionary history is just a hypothesis, and other evolutionary origin possibilities cannot be completely excluded, e.g. ancient origin of the P-DUDES domain followed by the widespread gene loss, or the independent gain of this domain in bacteria and vertebrates.

## Conclusions

It is always a challenge to turn a structure prediction into a function prediction with specific experimental validation suggestions. It is even a greater challenge to extend a molecular function prediction into a biological process prediction. The former is usually linked to protein structure, the latter not much so. Here, we endeavoured to complement structure predictions with the analyses of phylogeny, microbial habitat and lifestyle, and disease-related expression datasets in order to gain insight into the possible roles of the peroxiredoxin function postulated for P-DUDES genes. Nevertheless, the ultimate answers will only be provided by experiments, both biochemical and biological. The former will involve validation of the molecular peroxiredoxin function, including substrate identification, the latter may employ functional analyses, e.g. knock-down and overexpression analyses combined with various stimuli.

## Methods

### Identification of the P-DUDES domain, structure prediction, sequence analysis

For remote homology identification, PSI-BLAST searches were executed using the standard parameters on the nr and env_nr databases at NCBI as of January 2009. Saturated BLAST[23] searches used five iterations of PSI-BLAST on nr and env_nr databases, BLAST expect value 0.001 and redundancy threshold for selection of representative sequences set to 60% identity as criteria for seed selection. For Pfam domain assignments, HMMER2 on the Pfam database as of January 2009 were used. The P-DUDES proteins were re-checked using HMMER3 on the Pfam database as of October 2009.

For survey of similarities within the thioredoxin-like clan, the CLANS algorithm[29] was ran on a set of sequences including a) all the Pfam "seeds" from the 27 families of the clan (CL0172), b) the 145 representative P-DUDES domains, c) all the Pfam "seeds" from the Pfam families DUF929, DUF1094, DUF1462, DUF2847, DUF1223, ArsD, DUF2703, DUF3088 and TrbC_Ftype that were assigned by us to the CL0172 clan. CLANS was run with five iterations of PSI-BLAST, using the BLOSUM45 substitution matrix and inclusion threshold 0.001. For the graph, similarity relations with significance of *p*-value less than 0.001 were considered. Transmembrane region predictions were achieved by the TMHMM and MEMSAT servers[70,71]. The Jpred and PsiPred servers were used to predict the secondary structure of the P-DUDES domains[72,73]. Multiple alignments of the P-DUDES domain were built using the PROMALS[74] and MUSCLE programs[75]. The former includes the predicted secondary structures in the buildup of the alignment, and both employ remote homologues of the aligned sequences. For the multiple alignments, N- and C-terminal regions that could not be aligned to human P-DUDES proteins were removed from the bacterial P-DUDES proteins.

For structural prediction, the three methods used were FFAS3[76], that uses sequence profile-to-profile comparison, HHPRED[28] that employs HMM-to-HMM comparison, and Phyre[77], a metaserver that employs a number of prediction methods.

Phylogenetic analyses were performed using the phylogeny.fr server[78], employing the maximum likelihood method PhyML, with the Approximate Likelihood-Ratio Test (aLRT) for branch support. The trees were constructed using Muscle multiple sequence alignments for: a) human and *Danio rerio *P-DUDES domains, b) for the set of 145 representative P-DUDES domains supplemented by all *Gallus gallus *and *D. rerio *domains. The sequence variability was displayed as sequence logos using the WebLogo server[79]. Disordered structures were predicted by the DisEMBL server[38].

### Structure models of the P-DUDES domain

Sequence alignments produced by the FFAS03 structure prediction method were used to construct 3D structure models by the program MODELLER[80] using the standard procedures: *multichain *for dimers and *model single *for monomers. The models were energy-minimised using the Schrodinger OPLS_2005 force-field. Out of seven models presented by MODELLER, the one with most favourable *molpdf *score was selected for further analysis. The MetaMQAP server[81] was used to estimate the correctness of a 3D model using a number of mode quality assessment methods in a meta-analysis.

For the calculation of molecular surfaces, electrostatic potentials, and lipophilic potentials, the Vasco program was used using standard parameters[46]. Accessible surfaces were calculated using the Proface server[82]. For the analysis of conservation of surface features, the Consurf server was used[83], using five PSI-BLAST iterations, homologue detection from Uniprot, and a maximum of 100 homologues.

### Analysis of microbial P-DUDES domains

Habitats and lifestyles of bacterial P-DUDES protein-possessing organisms were collected from the Microbial Genomes resource within Entrez Genome Project database at NCBI. Analysis of genome environments of the P-DUDES bacterial species was performed using SEED[84,85], MicrobesOnline[86] and Integrated microbial genomes (IMG)[87]. Gene co-expression was studied using the STRING[53] tool.

### Expression and biological relationship analysis

For the identification of microarray experiments with similar expression patterns of human P-DUDES genes, the GeneChaser tool was used[54]. The chosen glioblastoma-related microarray gene expression datasets (GDS1813, GDS1962[55,56]) were downloaded from the Gene Expression Omnibus database at NCBI[88]. Genes in the GDS1813 dataset were filtered by requiring a "present" flag in at least 30 out of 52 samples. For the calculation of fold changes and genes correlated to the P-DUDES genes, as well as for gene expression data visualizations, Tibco Spotfire DecisionSite for Functional Genomics software was used (Tibco, Palo Alto, CA, USA, http://spotfire.tibco.com/). Fold changes were defined as ratios of mean expression values for glioblastoma versus normal brain samples. The significance of fold changes was estimated by the Student's *t*-test. In Fig. S7 [Additional file 1: Suppl. Fig. S7], expression fold change values above 2 or below 0.2, and *t*-test *p*-values better that 0.01 were required for both the comparisons.

## List of abbreviations

HGT: Horizontal Gene Transfer; Thx: Thioredoxin; EMT: epithelial-to-mesenchymal transition.

## Authors' contributions

MG discovered the P-DUDES similarity to peroxiredoxins. MG, KP, AM, TS, and AL carried out the bioinformatics analyses. MG, AG and KP conceived the study, and participated in its design and coordination. MG and KP drafted the manuscript. All authors read and approved the final manuscript.

## Supplementary Material

Additional file 1 **"PDUDES_Suppl_File" contains Supplementary Figure legends and all seven Supplementary Figures (S1-S7)**. Supplementary Fig. S1. Phylogenetic tree of representative vertebrate P-DUDES domains. Supplementary Fig. S2. Sequence alignment of representative P-DUDES proteins together with selected known peroxiredoxins. Supplementary Fig. S3. Sequence logo for selected P-DUDES domain regions. Supplementary Fig. S4. Surfaces near putative peroxidatic cysteine residue coloured by lipophilic potential or by electrostatic potential. Supplementary Fig. S5. Putative interaction surfaces of the P-DUDES domains from human SRPX, SRPX2, CCDC80 proteins, coloured by lipophilic potential or by electrostatic potential. Supplementary Fig. S6. Putative interaction surfaces of the P-DUDES domain from the human SRPX protein, coloured by sequence conservation among homologues. Supplementary Fig. S7. P-DUDES gene expression changes for two glioblastoma datasets. Click here for file
