# Supplementary material for: A widespread peroxiredoxin-like domain present in tumor suppression- and progression-implicated proteins
Source: BMC Genomics. 2010 Oct 21;11:590. doi: 10.1186/1471-2164-11-590 (PMC3091736; doi:10.1186/1471-2164-11-590)
Supplement: Additional file 1 — "PDUDES_Suppl_File" contains Supplementary Figure legends and all seven Supplementary Figures (S1-S7). Supplementary Fig. S1. Phylogenetic tree of representative vertebrate P-DUDES domains. Supplementary Fig. S2. Sequence alignment of representative P-DUDES proteins together with selected known peroxiredoxins. Supplementary Fig. S3. Sequence logo for selected P-DUDES domain regions. Supplementary Fig. S4. Surfaces near putative peroxidatic cysteine residue coloured by lipophilic potential or by electrostatic potential. Supplementary Fig. S5. Putative interaction surfaces of the P-DUDES domains from human SRPX, SRPX2, CCDC80 proteins, coloured by lipophilic potential or by electrostatic potential. Supplementary Fig. S6. Putative interaction surfaces of the P-DUDES domain from the human SRPX protein, coloured by sequence conservation among homologues. Supplementary Fig. S7. P-DUDES gene expression changes for two glioblastoma datasets. [file 1471-2164-11-590-S1.PDF]

Supplementary Figure Legends

Supplementary Fig. S1.

Phylogenetic tree of representative vertebrate P-DUDES domains

The phylogenetic tree was built using PhyML, and contains representative vertebrate P-DUDES domains (including five human domains and eleven *Danio rerio* domains)
For clarity, protein names are shortened: "_H" and "_D" suffixes denote human and *D. rerio* domains, respectively. Also for clarity, the two *D. rerio* paralogues of CCDC80 are referred to as "CCDC80-like 1" and "CCDC80-like 2". Suffixes "-1", "-2", "-3" denote 1st, 2nd and 3rd P-DUDES domains of CCDC80 proteins, respectively. The NCBI gi identifiers for the sequences are as follows: CCDC80_H [GenBank:74712933], SRPX2_H [GenBank:74739594], SRPX_H [GenBank:2498958], SRPX_D [GenBank:62122833], SRPX2_D [GenBank:189528097], CCDC80_D [GenBank:55742450], CCDC80L1_D [GenBank:189520212], CCDC80L2_D [GenBank:125847898].

Supplementary Fig. S2.

Sequence alignment of representative P-DUDES proteins together with selected known peroxiredoxins.

The sequence alignment, built using Muscle, includes 73 representative P-DUDES proteins together with selected known peroxiredoxins added, among them the templates used in structure modeling. Secondary structure, as determined in the 1we0 structure, indicated.

Supplementary Fig. S3.

Sequence logo for selected P-DUDES domain regions

Top logo – region of the presumed peroxidatic cysteine residue. Bottom logo – the beta-turn between strands β-3 and β-4.

Supplementary Fig. S4.

Surfaces near putative peroxidatic cysteine residue coloured by lipophilic potential or by electrostatic potential.

The peroxidatic cysteine residue is shown as yellow sticks, the surface is coloured by (a) lipophilic potential (red: lipophilic, blue: hydrophilic) or (b) by electrostatic potential (red: positive, blue: negative). All five human P-DUDES domains shown.

Supplementary Fig. S5.

Putative interaction surfaces of the P-DUDES domains from human SRPX, SRPX2, CCDC80 proteins, coloured by lipophilic potential or by electrostatic potential.

Putative interaction surfaces, (a-b) “type-A” (2ywi template) and (c-d) “type- B” (1we0 template) of the P-DUDES domains from human SRPX, SRPX2, CCDC80 proteins, coloured by (a, c) lipophilic potential (red: lipophilic, blue: hydrophilic) or (b,d) by electrostatic potential (red: positive, blue: negative). All five human P-DUDES domains shown.

Supplementary Fig. S6.

Putative interaction surfaces of the P-DUDES domain from the human SRPX protein, coloured by sequence conservation among homologues.

Putative interaction surfaces, (a)“type-A” (1xvw template), (b) “type-A” (2ywi template), (c). “type-B” (1we0 template) of the P-DUDES domain from the human SRPX protein, coloured by sequence conservation among homologues (Consurf algorithm). magenta: high conservation; blue: high variability; green: cysteines.

Supplementary Fig. S7.

P-DUDES gene expression changes for two glioblastoma datasets.

Expression changes for the three human P-DUDES genes in two glioblastoma datasets. 1032 genes visible with significant and large expression fold changes (FC) between glioblastoma and normal brain samples in both microarray experiments (see Methods for details).

Suppl. Fig. S1

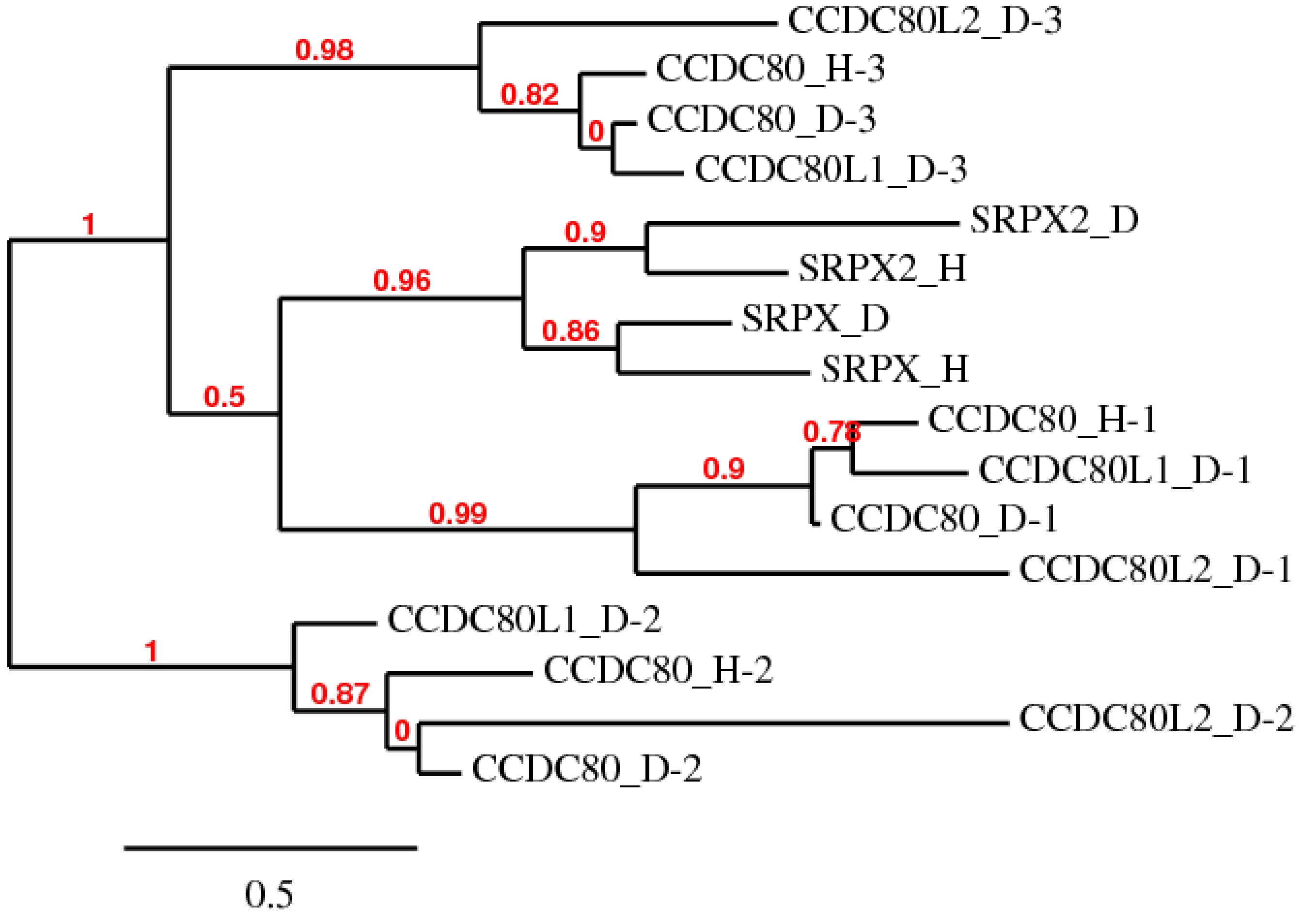
CCDC80L2_D-3
CCDC80_H-3
CCDC80_D-3
CCDC80L1_D-3
SRPX2_D
SRPX2_H
SRPX_D
SRPX_H
CCDC80_H-1
CCDC80L1_D-1
CCDC80_D-1
CCDC80L2_D-1
CCDC80L1_D-2
CCDC80_H-2
CCDC80L2_D-2
CCDC80_D-2
0.98
0.82
0
1
0.9
0.96
0.86
0.5
0.78
0.9
0.99
1
0.87
0
0.5

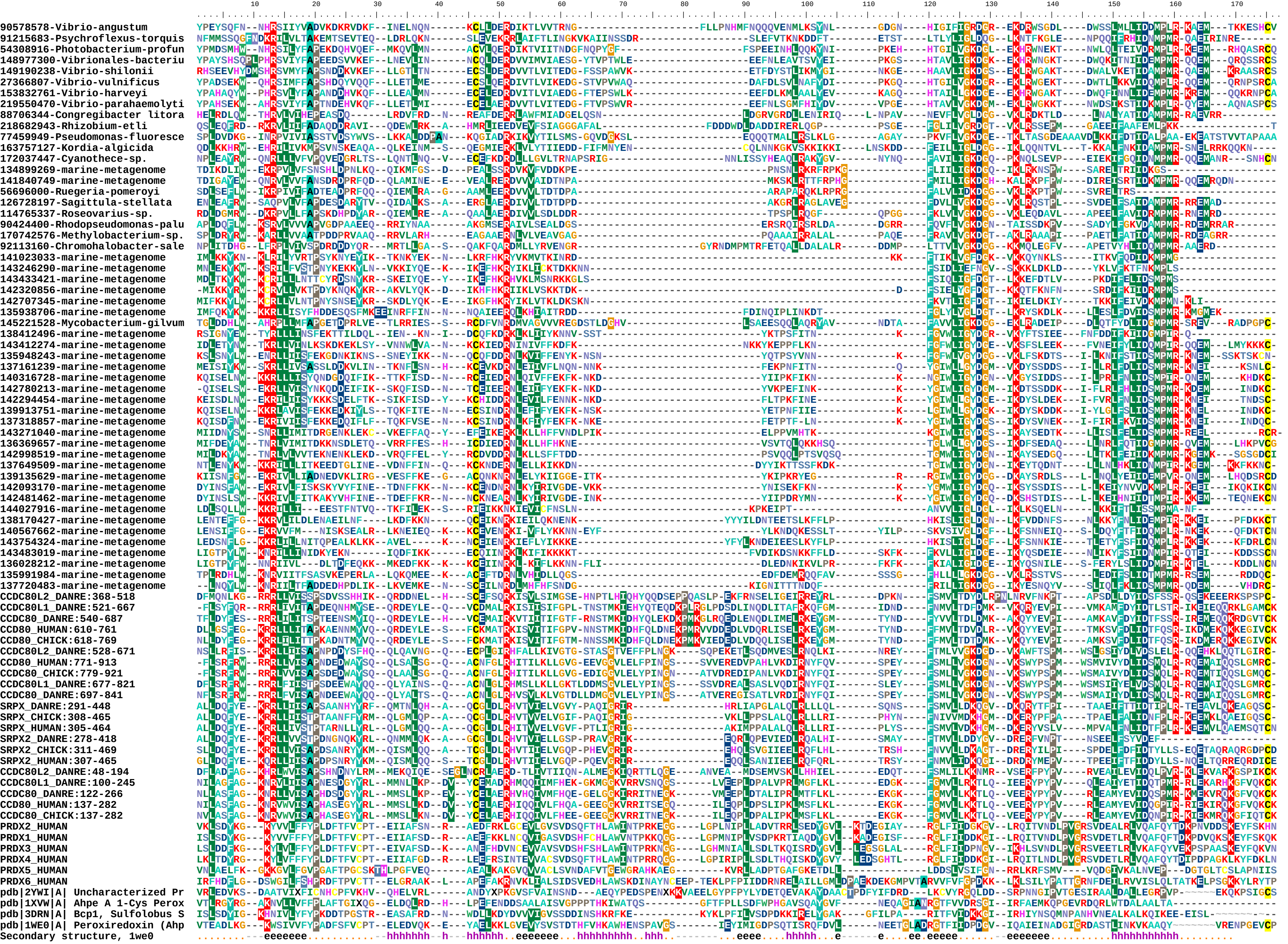

Suppl. Fig. S2

Suppl. Fig. S3

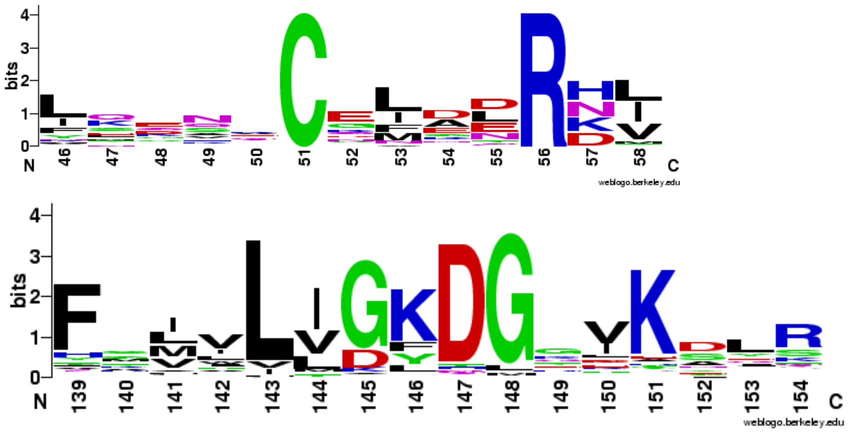
bits
N
46 47 48 49 50 51 52 53 54 55 56 57 58
C
weblogo.berkeley.edu
bits
N
139 140 141 142 143 144 145 146 147 148 149 150 151 152 153 154
C
weblogo.berkeley.edu

Suppl. Fig. S4a

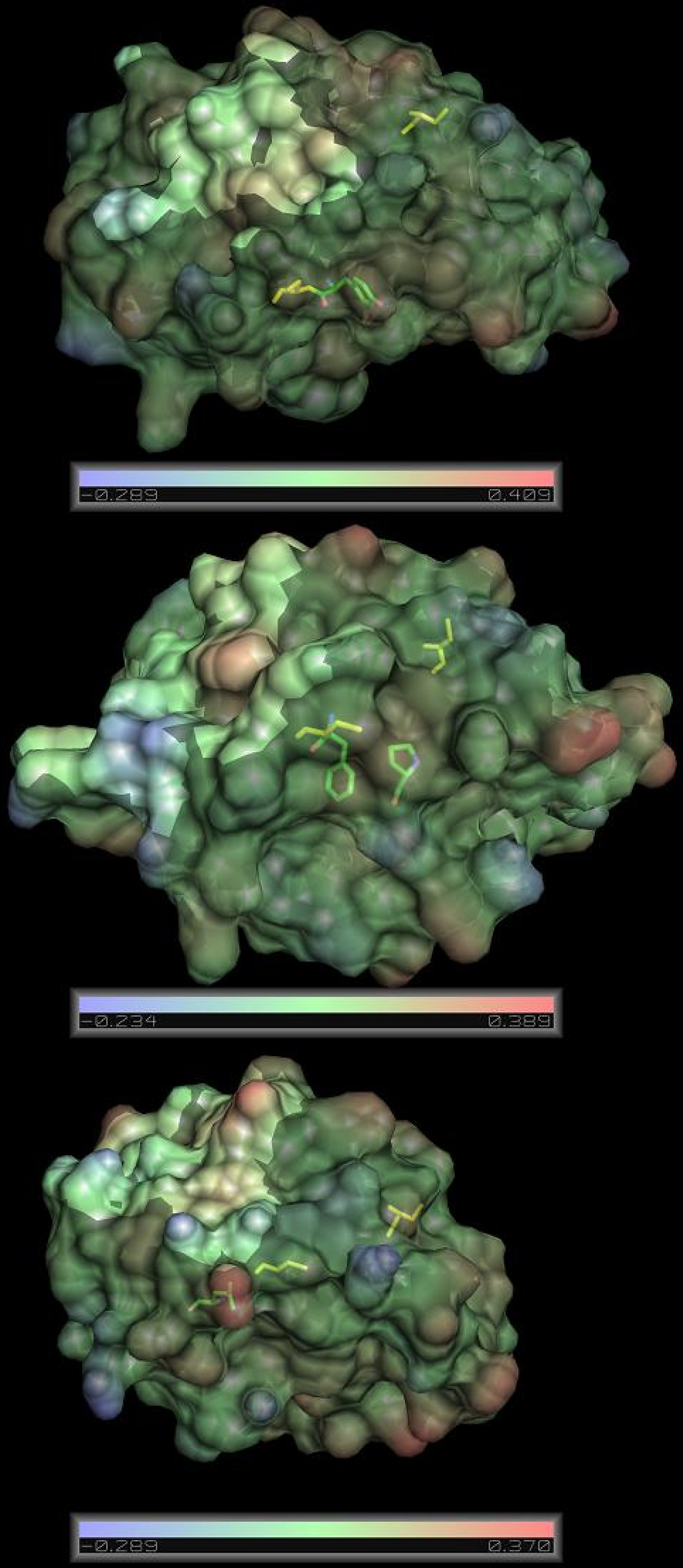

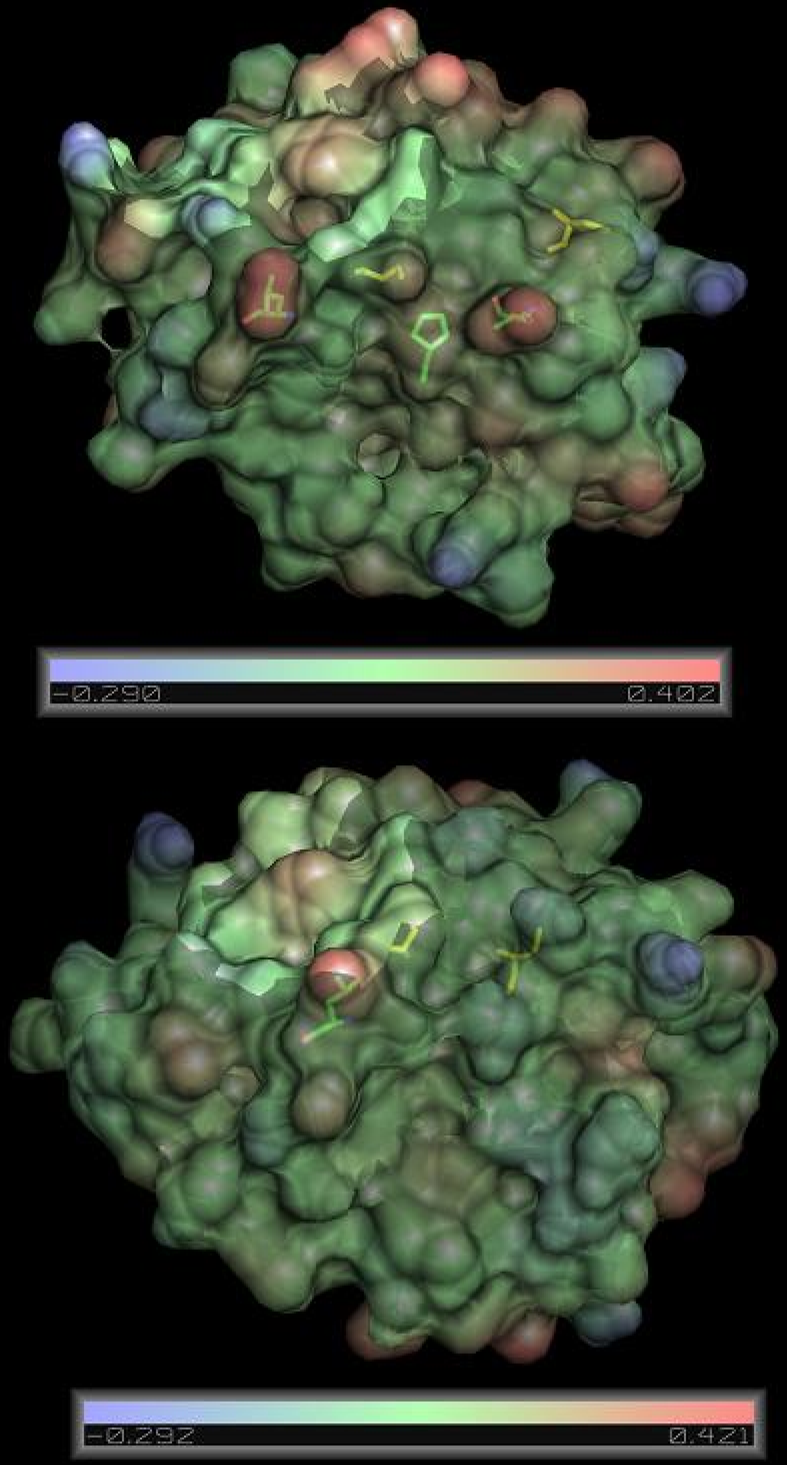

Suppl. Fig. S4b

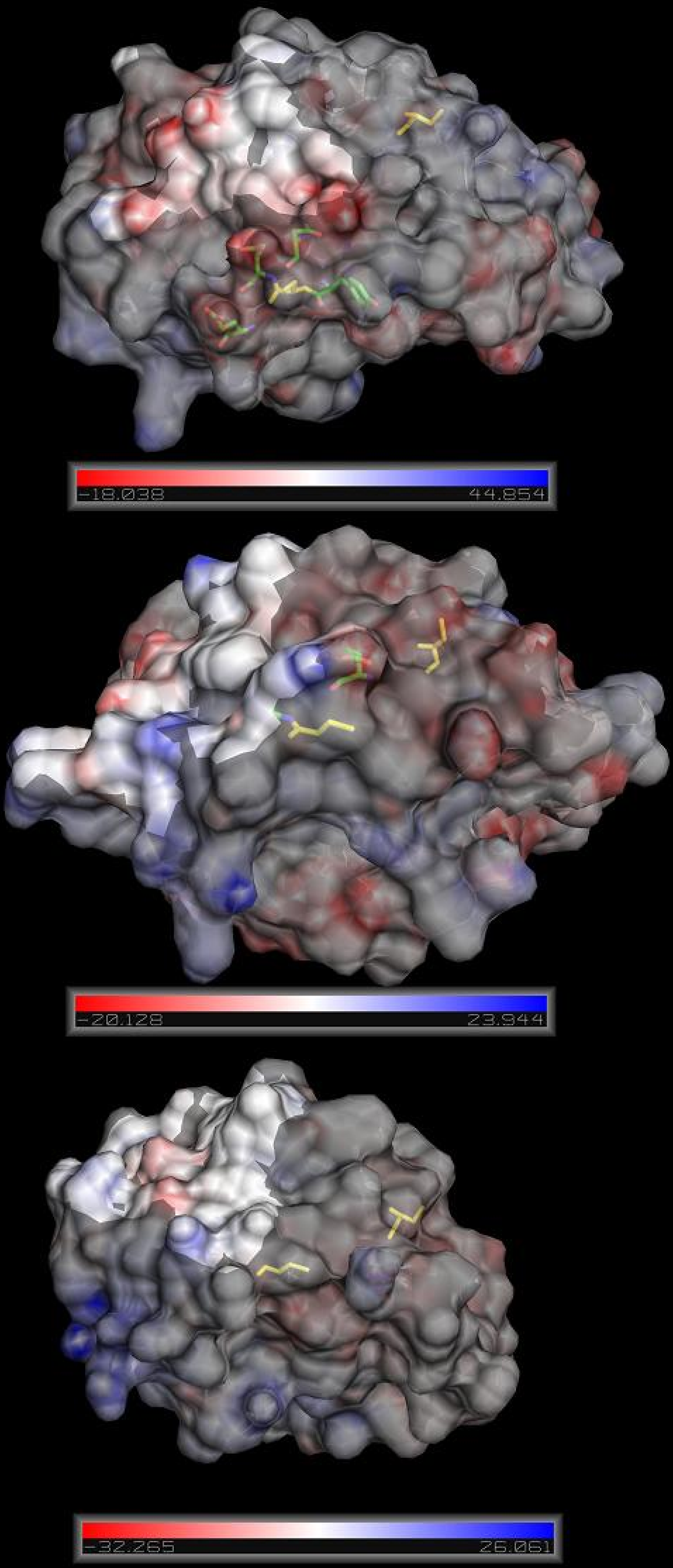

Suppl. Fig. S5a

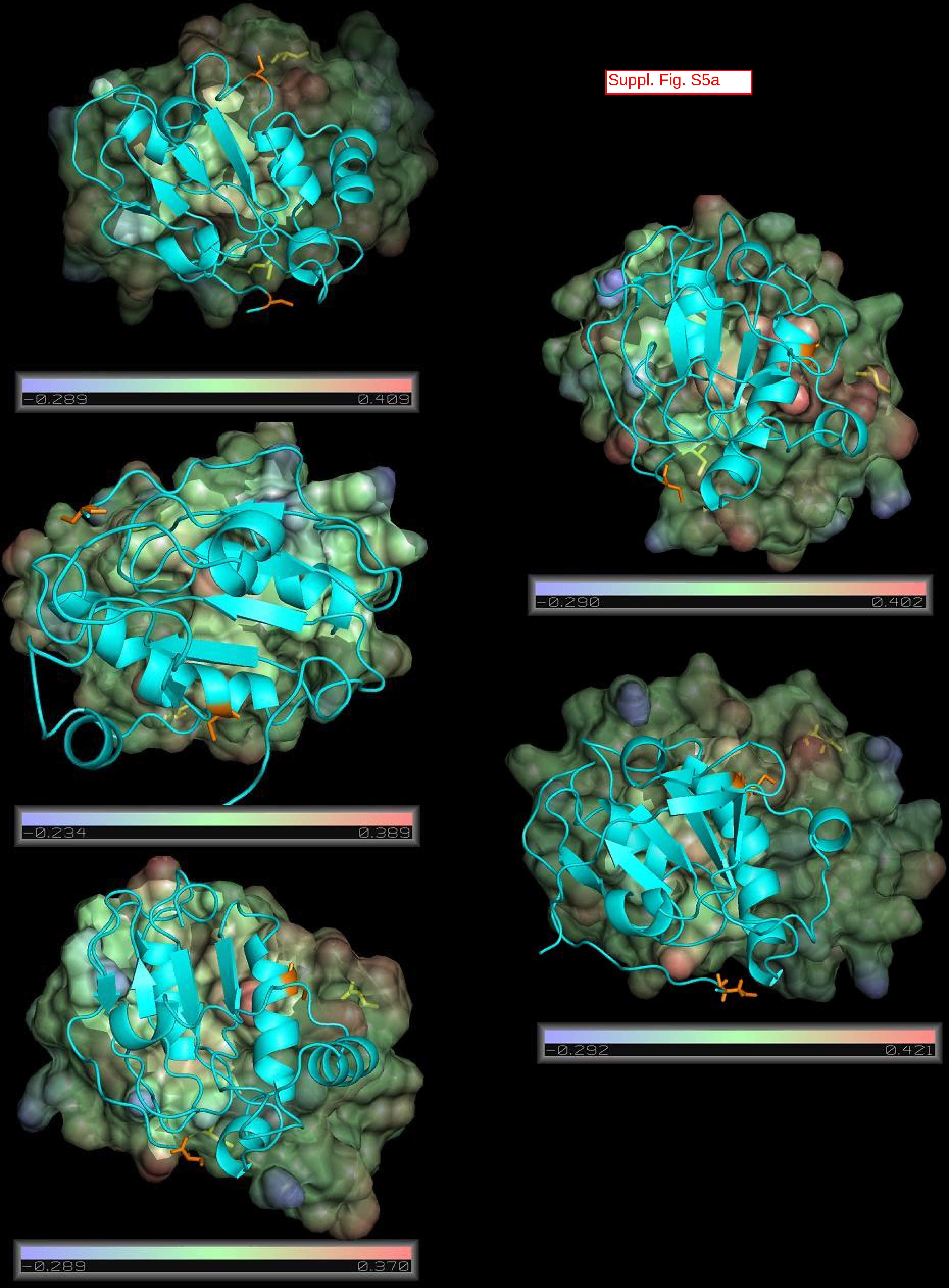

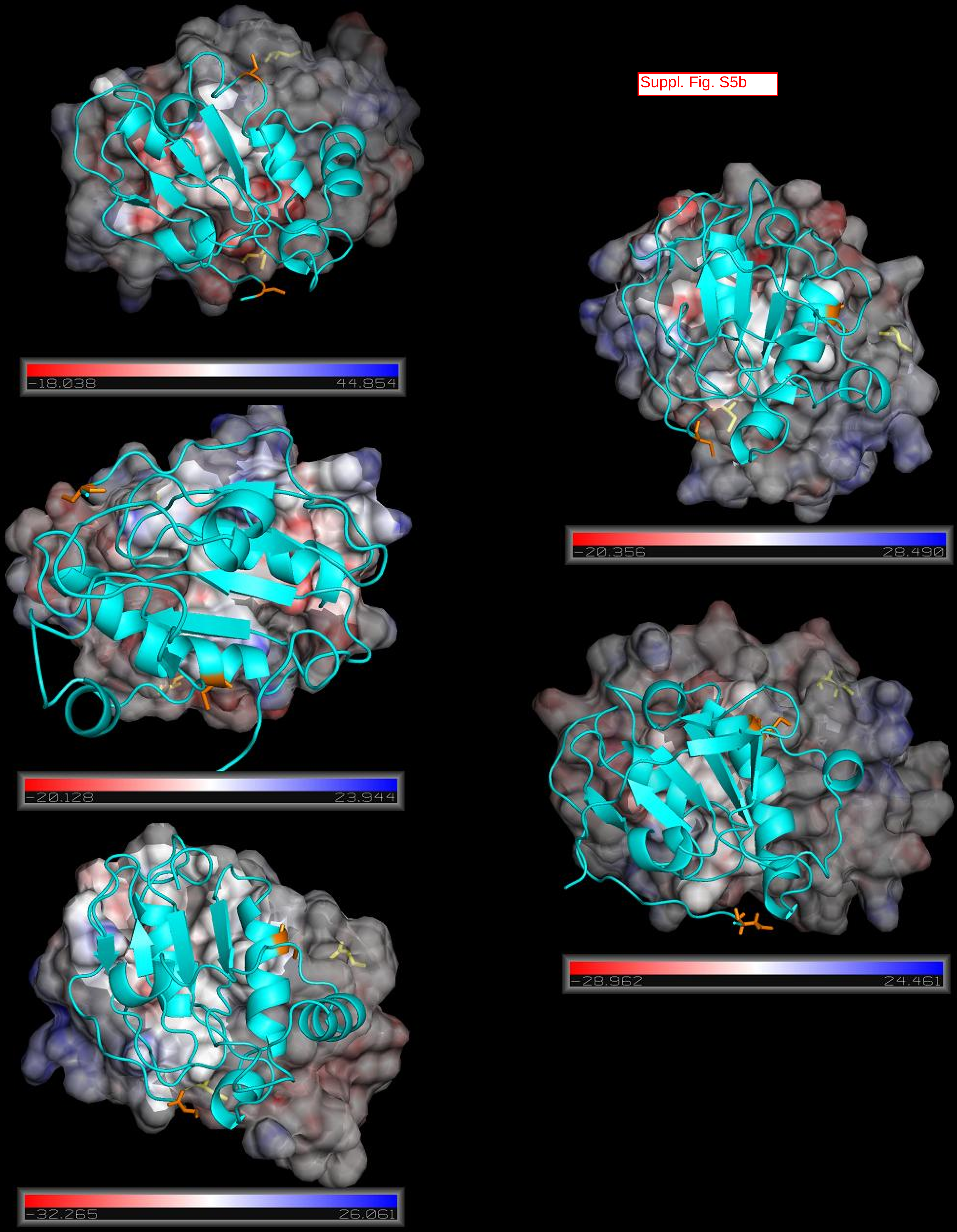

Suppl. Fig. S5b

Suppl. Fig. S5c

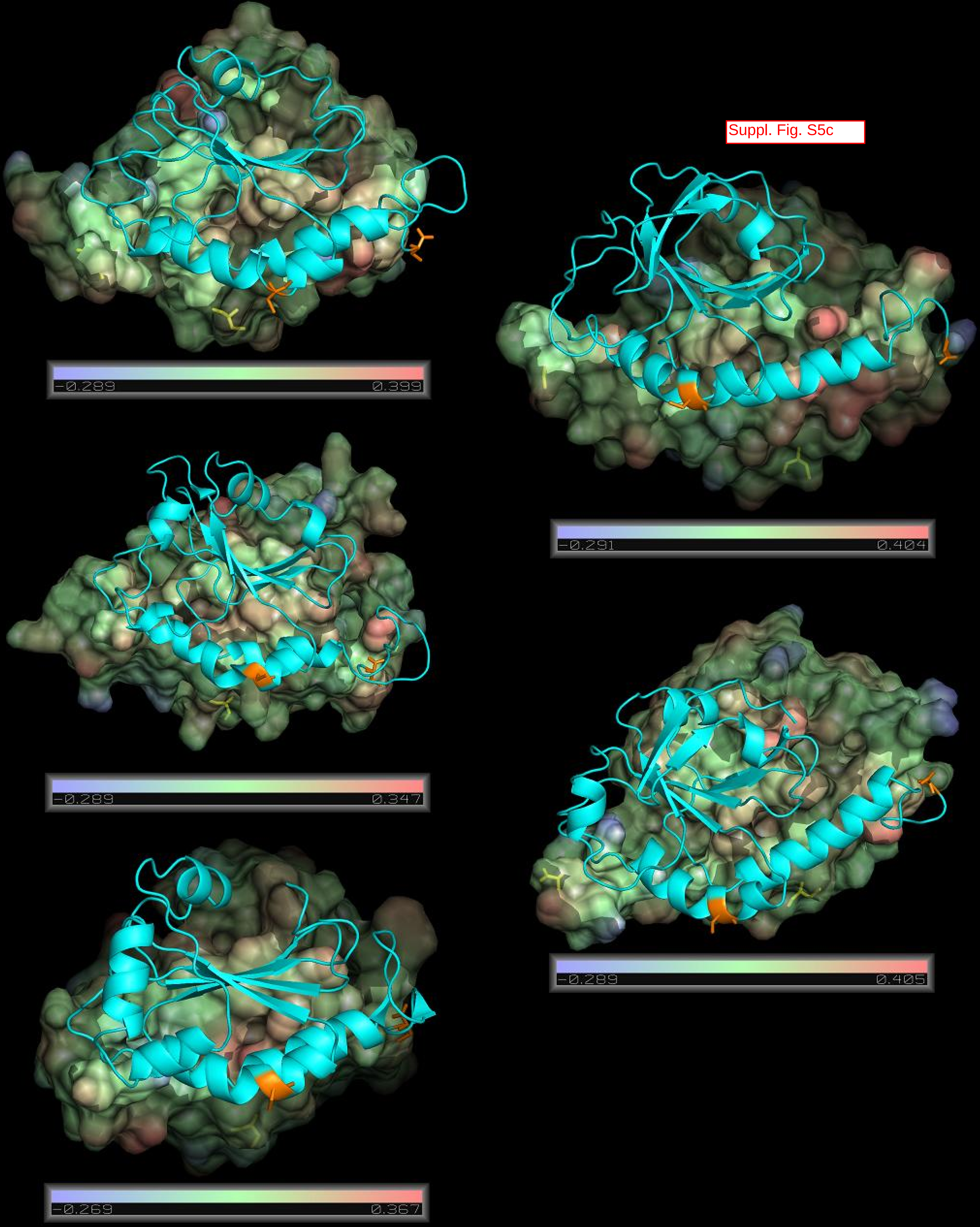

Suppl. Fig. S5d

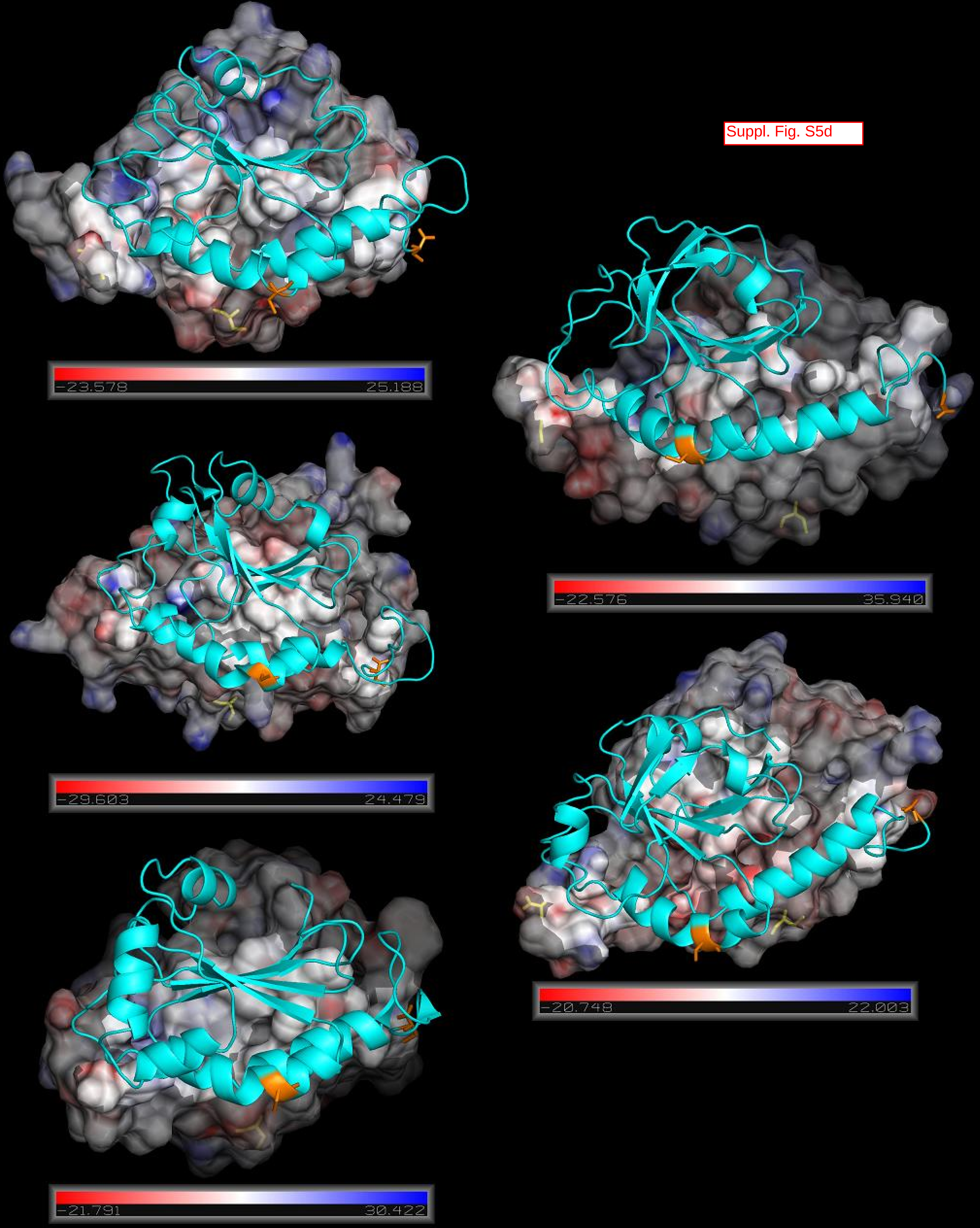

Suppl. Fig. S6

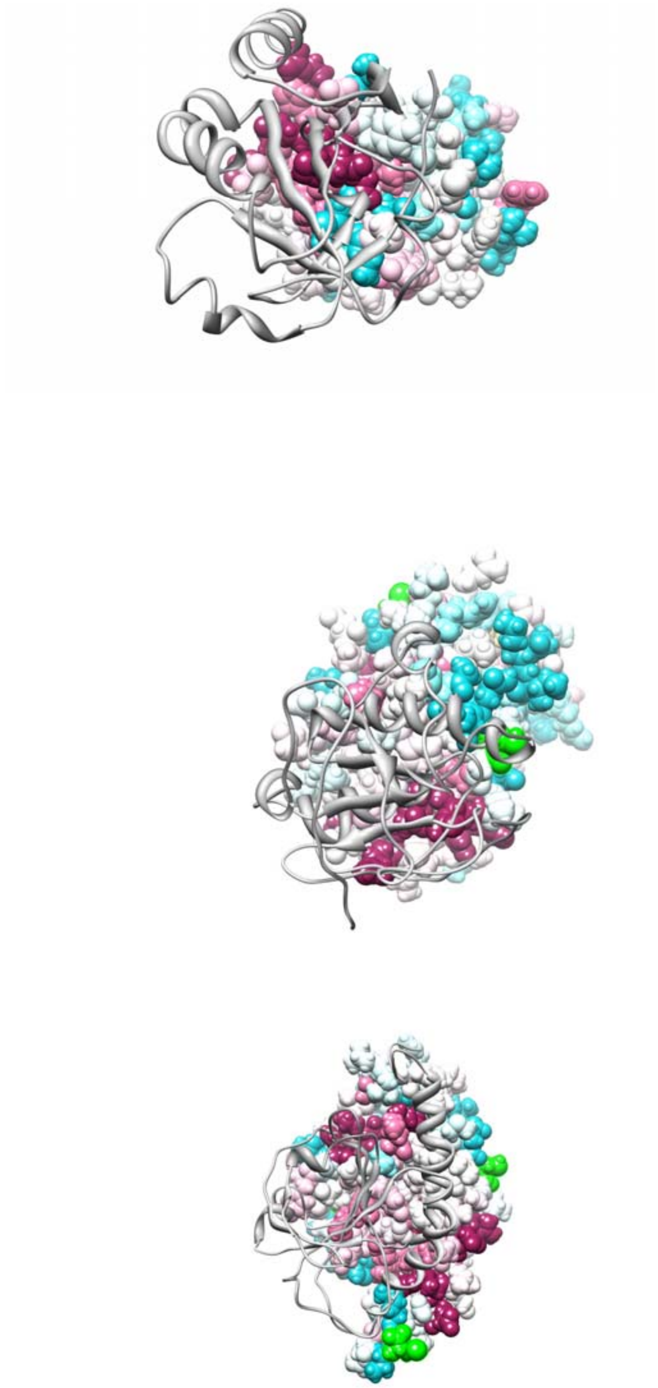

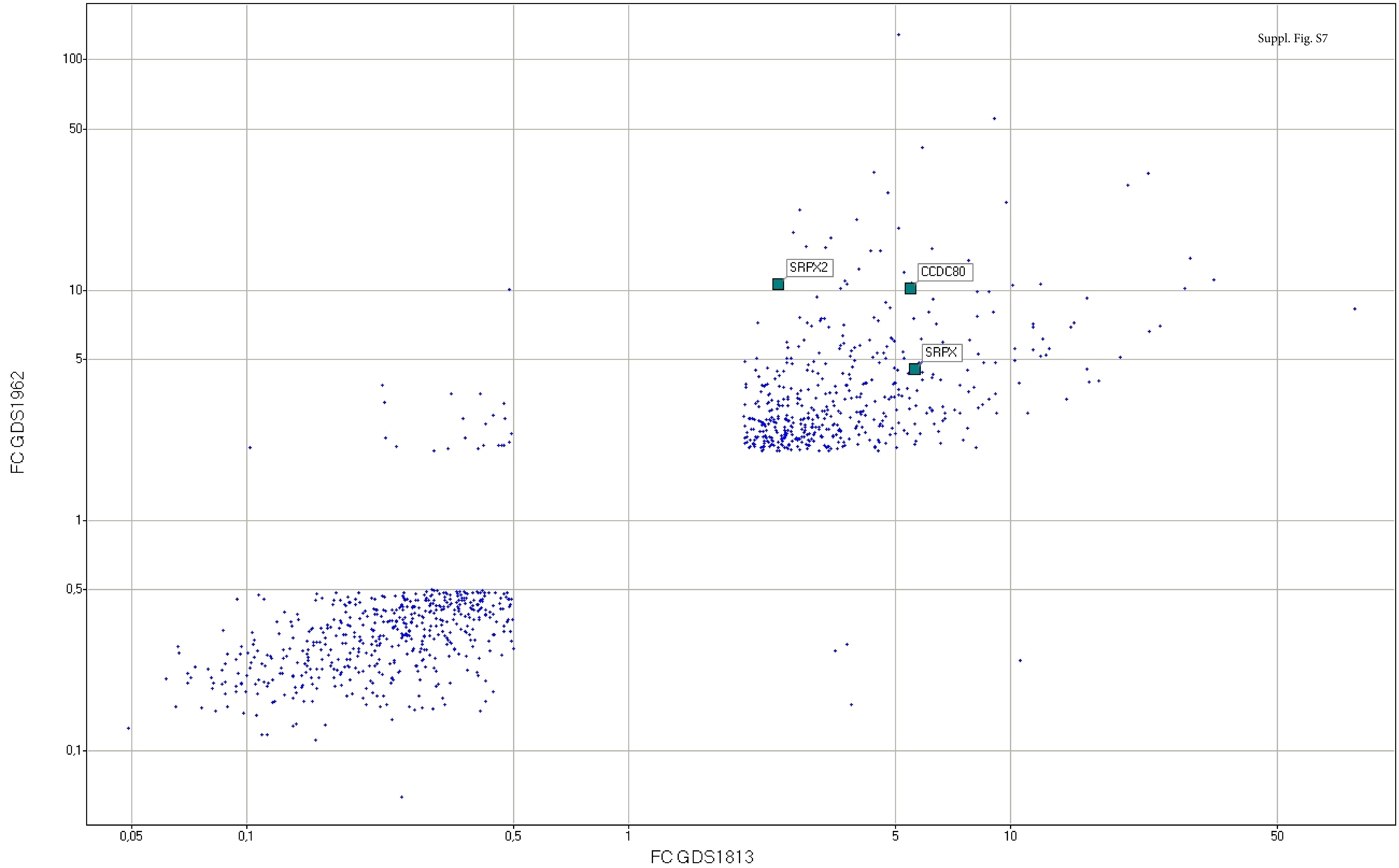

Suppl. Fig. S7
SRPX2
CCDC80
SRPX
100
50
10
5
1
0,5
0,1
FC GDS1962
0,05
0,1
0,5
1
5
10
50
FC GDS1813
